# Ionizing radiation induces transgenerational effects of DNA methylation in zebrafish

**DOI:** 10.1038/s41598-018-33817-w

**Published:** 2018-10-18

**Authors:** Jorke H. Kamstra, Selma Hurem, Leonardo Martin Martin, Leif C. Lindeman, Juliette Legler, Deborah Oughton, Brit Salbu, Dag Anders Brede, Jan Ludvig Lyche, Peter Aleström

**Affiliations:** 1 Faculty of Veterinary Medicine, Norwegian University of Life Sciences, 0033 Oslo, Norway; 2 University of Camagüey, Faculty of Agropecuary Sciences, Camagüey, 70100 Cuba; 3 Faculty of Environmental Sciences and Natural Resource Management, Norwegian University of Life Sciences, 1433 Ås, Norway; 4 Institute for Environment, Health and Societies, College of Health and Life Sciences, Brunel University London, Uxbridge, United Kingdom; 5 Utrecht University, Institute for Risk Assessment Sciences, 3508 TD Utrecht, The Netherlands

## Abstract

Ionizing radiation is known to cause DNA damage, yet the mechanisms underlying potential transgenerational effects of exposure have been scarcely studied. Previously, we observed effects in offspring of zebrafish exposed to gamma radiation during gametogenesis. Here, we hypothesize that these effects are accompanied by changes of DNA methylation possibly inherited by subsequent generations. We assessed DNA methylation in F1 embryos (5.5 hours post fertilization) with whole genome bisulfite sequencing following parental exposure to 8.7 mGy/h for 27 days and found 5658 differentially methylated regions (DMRs). DMRs were predominantly located at known regulatory regions, such as gene promoters and enhancers. Pathway analysis indicated the involvement of DMRs related to similar pathways found with gene expression analysis, such as development, apoptosis and cancers, which could be linked to previous observed developmental defects and genomic instability in the offspring. Follow up of 19 F1 DMRs in F2 and F3 embryos revealed persistent effects up to the F3 generation at 5 regions. These results indicate that ionizing radiation related effects in offspring can be linked to DNA methylation changes that partly can persist over generations. Monitoring DNA methylation could serve as a biomarker to provide an indication of ancestral exposures to ionizing radiation.

## Introduction

Transgenerational effects following ionizing radiation are well documented and include an extensive list of mostly stochastic effects. These include genomic instability, leading to tumour induction, changes in kinase activity and cell proliferation^1^, as well as effects on the endocrine system^2,3^. Since genomic instability is known to be inherited via non-Mendelian mechanisms, the origin of these transgenerational effects might to a certain extent be explained in underlying epigenetic marks^4,5^. As epigenetic changes can be transferred to offspring, they could play an important role in the aetiology of heritable effects related to ionizing radiation^6–9^. However, transgenerational changes on epigenetic marks following ionizing radiation exposure play an as yet poorly understood role^4^.

Epigenetic marks include non-coding RNAs, different histone isoforms and biochemical modifications on histones, and cytosine methylation (mC) in DNA^8^. Cytosine methylation has been studied most intensively in the context of transgenerational effects^10^. In vertebrates, mC (predominantly found at CpG dinucleotides) has been found to influence chromatin structure and gene expression^11^. Cytosine methylation is thought to be epigenetically inherited by multiple generations, with possible crosstalk between histone modifications and non-coding RNAs^8,12^. Transgenerational inheritance is shown by a number of studies, where changes of the methylome were found up to fourth generation in different animal studies following ancestral exposure to environmental contaminants^13–17^.

Currently, to our knowledge, there are no reports of transgenerational genome wide effects of DNA methylation following ionizing radiation exposures in vertebrates. Recently, in the invertebrate model *Daphnia magna*, transgenerational effects of DNA methylation following low dose radiation exposure were reported^18^. Furthermore, the potency of ionizing radiation to affect all epigenetic marks following direct exposure has been well documented. Most studies report ionizing radiation induced effects on DNA methylation in different *in vitro* and *in vivo* models on for instance global levels^19–23^, at repetitive elements^24–26^, and more focused with genome wide^27,28^, and locus specific analyses^20,29^. In a one generational study, effects on global methylation have been reported in progeny from mice exposed to ionizing radiation^30,31^. Although these results indicate the involvement of DNA methylation in ionizing radiation response, it is unclear if transgenerational effects of DNA methylation following ionizing radiation exposures are to be expected. Therefore, genome wide analysis of DNA methylation in a transgenerational set-up is warranted, to aid in the elucidation of ionizing radiation induced transgenerational effects.

Here, we aimed to study the relationship between changes in DNA methylation and gene expression and whether these changes were persistent over generations, using zebrafish as a model. Zebrafish is a widely used vertebrate model in both radiation^32^ as well as epigenetic research^33^, largely because of its conserved molecular pathways in DNA repair and epigenetics. Somatic DNA methylation patterns are similar in zebrafish compared to mammals, but DNA methylation reprogramming during early embryonic development differs^34,35^. In mammals, the parental genomes are demethylated in the early phases of embryonic development to ensure a pluripotent cell state^36^, whereas in zebrafish the paternal genome seems almost unaffected during this early stage^35^. The methylome is re-established before zygotic genome activation (ZGA) in zebrafish^34,35^, whereas in mice, this occurs beyond ZGA^37^. Still, the DNA methylation pathways as well as the tissue specific levels of hydroxymethylcytosine (hmC), an intermediate in the demethylation mechanism, are conserved^33,38^. Several studies have successfully employed zebrafish for DNA methylation studies following xenobiotic exposures^15–17,39–45^, however information of ionizing radiation induced epigenetic changes in zebrafish is scarce^46^.

We previously reported effects in F1 progeny derived one year after exposure of adult zebrafish (F0) for 27 days to 8.7 mGy/h, such as increased inflammation and genomic instability and defects in eye formation^47^. The effects could be linked to enriched pathways following gene expression analysis in 5.5 hpf F1 offspring^48^. In this study, we analysed DNA methylation in 5.5 hpf F1, F2 and F3 embryos from 8.7 mGy/h exposed ancestors one year after exposure, using whole genome bisulfite sequencing (WGBS) on F1 offspring. A selection of differentially methylated regions (DMRs) derived from WGBS was measured with locus specific analysis in F2 and F3 embryos. Our results indicate a relationship between overrepresented pathways of genes associated to DMRs and gene expression. Specific loci were persistently changed up to F3, indicating the involvement of DNA methylation on transgenerational effects caused by ionizing radiation.

## Material and Methods

### Zebrafish husbandry

This study was approved by the institutional animal ethics committee (IACUC) and the Norwegian food inspection authority (NFIA), under permit number 5793. Zebrafish (AB wt) were obtained from the NMBU zebrafish facility, licensed by the NFIA and accredited by the association for assessment and accreditation of laboratory animal care (AAALAC, license number: 2014/225976). The NMBU zebrafish facility and SOPs has AAALAC accreditation (No. 1036) and is approved by the National Animal Research Authority. All experiments were performed according to Norwegian Animal Welfare Act (2009) and the EU Directive 2010/63, following appropriate guidelines. Six month old males and females (F0) (N = 30) were kept at 28 ± 1 °C on a 14–10 hour light-dark cycle at a density of up to 10 fish/L. Housing conditions are described in detail in SI materials and methods.

### Exposure of zebrafish and generational set-up

**Figure 1:**
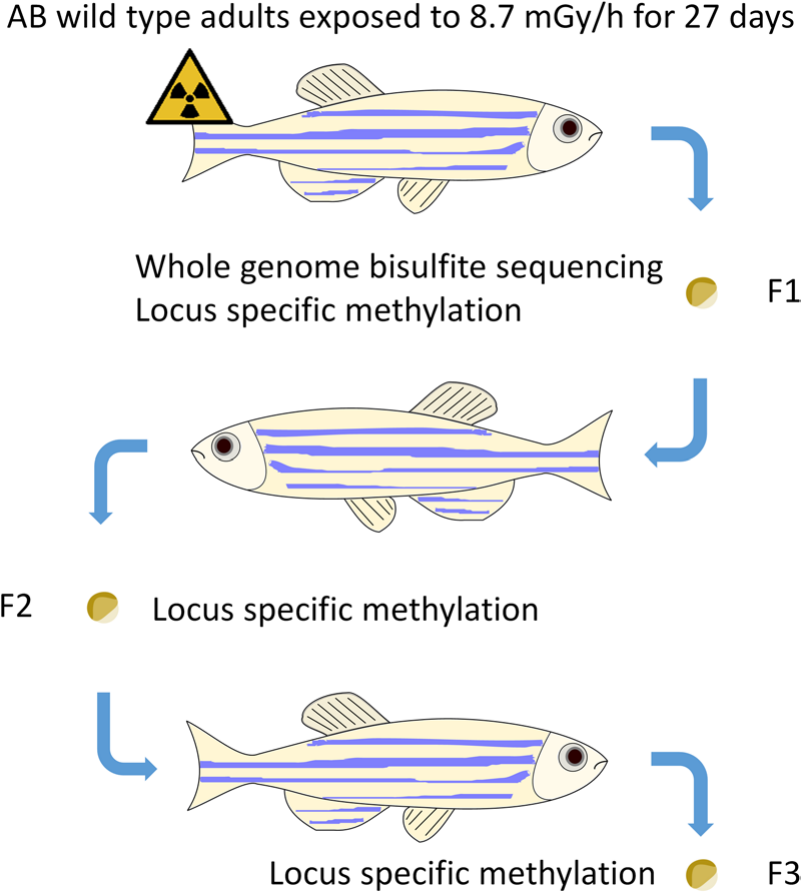
Experimental set-up. Family inbreeding was performed of control and exposed adults (8.7 mGy/h), embryos were harvested 5.5 hours post fertilization for DNA methylation analysis at every generation.

Full details of the parent fish irradiation and dosimetry can be found elsewhere^47^. In brief, the fish were irradiated at an average dose rate of 8.7 mGy/h for 27 days (total dose 5.2 Gy, Fig. 1) in 9 L plastic tanks at the ^60^Co irradiation facility (FIGARO, NMBU), maintained according to standard operating procedures of the NMBU zebrafish facility. The dose compares to observations from Chernobyl, where the total accumulated dose over 60 days post-accident was estimated at 10 Gy^49^. These doses did not cause effects on survival in our study on directly exposed adult zebrafish^50^. The swimming space volume was approximately 6 L (16 × 18 × 22 cm) with custom made grids. Dosimetry was calibrated by a combination of air kerma measurements (traceable back to national and international standards), tank dosimetry measurements and modelling of dose absorption and attenuation across the tanks^47^. Fish were maintained for one year before generating F1. Generations F1 and F2 were generated by family inbreeding^47^.

### Generation of embryos

DNA methylation was performed in 50% epiboly embryos (5.5 hpf, early gastrulation), in F1, F2 and F3 generation (Fig. 1). In order to obtain sufficient DNA for downstream analysis, we pooled eggs derived from 7 parallel 1 L breeding set-ups with 2 males and 3 females for each treatment at each generation. The pooled eggs were divided in portions of 100 eggs in 24 wells plates containing 2 mL autoclaved system water per well and incubated at 28 °C. At 2 hpf embryos were assessed for quality and unfertilized eggs and bad quality embryos were removed. Embryos were incubated up to 50% epiboly and snap frozen in liquid nitrogen and stored at −80 °C until further analysis.

### DNA purification

From each pool of 100 embryos, DNA was purified with the Puregene tissue DNA extraction kit (Qiagen, Germany), with modifications described in SI materials and methods. DNA was assessed for RNA contamination and integrity with gel electrophoreses and measured for concentration and quality by Nanodrop (ND-1000; Thermo Scientific). DNA was stored at −20 °C until further analysis.

### Whole genome bisulfite sequencing

We analysed 3 control and 3 exposed F1 DNA samples for DNA methylation with WGBS. From each sample, the DNA concentration was analysed by the Qubit system (Life technologies, Norway). To each sample, 0.1% lambda DNA was added to measure bisulfite conversion efficiency. Details of library preparation can be found in SI materials and methods. Following sequencing, generated fastq files were analysed for quality using Fastqc (v 0.11.5, Babraham Institute, UK). Fastq files were adapter trimmed with Trim Galore! (v 0.4.1, Babraham Institute, UK) with standard parameters, using a quality phred score cut-off of 20, with an extra base trimmed from 3′ side and 2 bases from the 5′ side from read 2. Trimmed sequences were mapped against the zebrafish genome (GRCz10) using Bismark (v 0.15, Babraham institute), using the stringent option -L 30 with slightly relaxed parameters for mapping (score-min L, 0, −0.4). Initial data analysis was performed by Seqmonk (v1.36, Babraham Institute). Differential methylation was assessed with methylKit (v1.0.0) in R (v3.3.1). DMRs were detected using logistic regression with a sliding linear model (SLIM)^51^. With methylKit we used a 99.9% percentile cut-off in read depth, in order to exclude areas with extremely high read depths due to either PCR bias or mapping to repeat regions. We generated 250 bp tiles with a sliding window of 125 bp and ran methylKit analysis on tiles with at least 5 mutually analysed Cs with at least 5 reads per C. Tiles with at least 10% difference in methylation and a Q value of 0.01 were considered significant. Raw sequencing data and detailed data files of all analyses can be found under Gene Expression Omnibus (GEO) number GSE100470.

### Detection of overrepresented clusters of differentially methylated regions and differentially expressed genes

We used our previously published list of differentially expressed genes from the same batch of F1 embryos as described in this study (accession number: GSE98539). Clustering of genomic regions was performed as previously described^52^. Window sizes were used of 2 Mb with 50 kb sliding windows, counting the number of DMRs or differentially expressed genes (DEGs) at each location. Z-scores were calculated for each sliding window, which were converted to a P value. A cluster was created by using a P value of 0.05 or lower, and overlapping consecutive significant windows were merged. A background model was generated by performing the same analysis on all measured tiles or all annotated genes for DNA methylation and RNA seq analysis, respectively.

### ATAC and ChIP bioinformatics

We used previously published data sets to associate the observed DMRs to different histone post translational modifications and accessible chromatin (ATAC). We used ATAC seq (PRJCA000283)^53^, histone H3 lysine 4 trimethylation (H3K4me3), H3K4me1, H3K27ac (GSE:32483)^54^, H3K27me3 and H3K36me3 (GSE44269)^55^ data from dome stage (4.3 hpf). After adapter and quality trimming with Trim galore! (v0.4.1, Babraham Institute, UK), Bowtie2 (v2.2.9) was used for mapping the sequence data to the GRCz10 assembly. Bam files were loaded in Seqmonk (v1.36) and log2 normalized read counts were measured 5 kb up and downstream of DMRs.

### Gene nomenclature

We used gene nomenclature adapted to the repositories of the different analyses. For Ingenuity Pathway Analysis (IPA, Qiagen), results are based on human pathway and regulators, and therefore we kept the human protein nomenclature output from IPA (e.g. HNF4A). This was also done for the transcription factor enrichment. For all other analyses we refer to the zebrafish gene nomenclature (*hnf4a*).

### Pathway analysis

In order to associate DMRs to genes we used the genomic regions of enrichment annotation tool (GREAT)^56^, using the standard parameters. First an initial regulatory domain is defined as 5 kb upstream and 1 kb downstream of the transcriptional start site (TSS) of a gene. This is extended with 1,000 kb in both directions, up to the initial regulatory domain of the nearest gene. The resulting gene list was imported in Webgestalt (http://www.webgestalt.org/), for KEGG pathway analysis, using hypergeometric calculations with Benjamini Hochberg corrections to adjust P values for multiple comparisons. Within IPA, the GREAT gene list was used to search for enrichments in pathways, upstream regulators and diseases, using the core analysis. IPA uses Fisher exact tests to calculate overrepresentation. IPA uses homologene (https://www.ncbi.nlm.nih.gov/homologene) to search for human orthologues of zebrafish genes. Since we used WGBS, all genes could be associated with measured DNA methylation, hence we used the complete gene annotation as background in the overrepresentation analyses.

### Motif enrichments and transcription factor analysis

MEME-ChIP is designed for scanning genomic regions from large scale DNA data-sets for motifs^57^. We focused on the results from the MEME algorithm within MEME-ChIP. In order to investigate if the discovered motifs were similar to consensus sequences of known transcription factors (TFs) we used Tomtom which compares the discovered motifs against databases (Eukaryote DNA) and calculates scores based on similarities in sequences, which is converted to a P value (<0.01 was considered significant)^58^.

### Locus specific DNA methylation

We selected 20 target from the methylKit analysis to validate the WGBS data and analysed these targets in F2 and F3 generation embryos (Supplemental Figure S1). One target (*atm*) did not show consistent results following standard curve analysis and was discarded. We used 5 replicate pools of embryos per exposure group per generation with this analysis. We used the BisPCR2 method^59^, which was adapted at our lab and is extensively described^16^, and details can be found in SI materials and methods. Primers were developed using the online Bisearch tool (http://bisearch.enzim.hu/), and validated for specificity and amplicon size by gel electrophoresis, as described previously (Supplemental Table S1)^16^. Each primer was validated with standard curve analysis using different ratios of unmethylated DNA to fully methylated DNA. Unmethylated DNA was produced by means of whole genome amplification (Qiagen, Germany) and methylated DNA by M.SssI methyltransferase (New England Biolabs, US)^16^. Downstream bioinformatics analysis was performed similarly as with WGBS. Statistical analysis was performed with Seqmonk (v1.36), using the logistic regression filter, with Benjamini-Hochberg FDR correction, with a methylation cut-off of 10%.

## Results

### Quality Control

In order to obtain sufficient DNA for WGBS analysis from a relative homogenous cell population, we targeted the early gastrula stage, at 50% epiboly. This stage provides information from both maternal and paternal ancestral exposures, and has limited bias in changes of cell type populations as shown in recent single cell transcriptomic analysis^60^, which could be apparent at later developmental stages due to increasing numbers of differentiated cell types showing segregated phenotypes^61^. With our previous published RNA sequencing data set we performed a global assessment of increases in mutation rates by assessing insertions, deletions and chimeric reads in exons and found no significant increases in mutation rates^48^.

With WGBS, we analysed three replicates of zebrafish embryos derived from F0 control and exposed adults (8.7 mGy/h for 27 days, total dose 5.2 Gy, hereafter referred to as exposed samples) for DNA methylation analysis using >120 million sequences per sample (Supplemental Table S2). General mapping statistics to the GRCz10 assembly of zebrafish showed an average mapping efficiency of 74.4%, which resulted in over 43 million cytosines in CpG context covered with at least 1 read (>90% of all CpGs), showing an average methylation of cytosines in CpG context of 80.7% (Supplemental Table S2). Lambda DNA was added to each sample to assess bisulfite conversion efficiency, which was higher than 98%, except for one exposed sample (95.6%) (Supplemental Table S2).

For the locus specific analysis we performed a standard curve analysis for each loci, with 0% to 100% methylated DNA, where 19 out of 20 targets showed a linear relationship. The region located in the promoter of the *atm* gene did not show a linear relationship and was discarded from the analysis. Most of the targets did not reach 100% methylation (Supplemental Figure S2). Since, this was consistent with many targets and also observed in our previous study^16^, we hypothesize that the *in vitro* CpG methylation was not efficient enough to reach 100% methylation at some loci.

### Differentially methylated regions are preferentially located at regulatory regions

**Figure 2:**
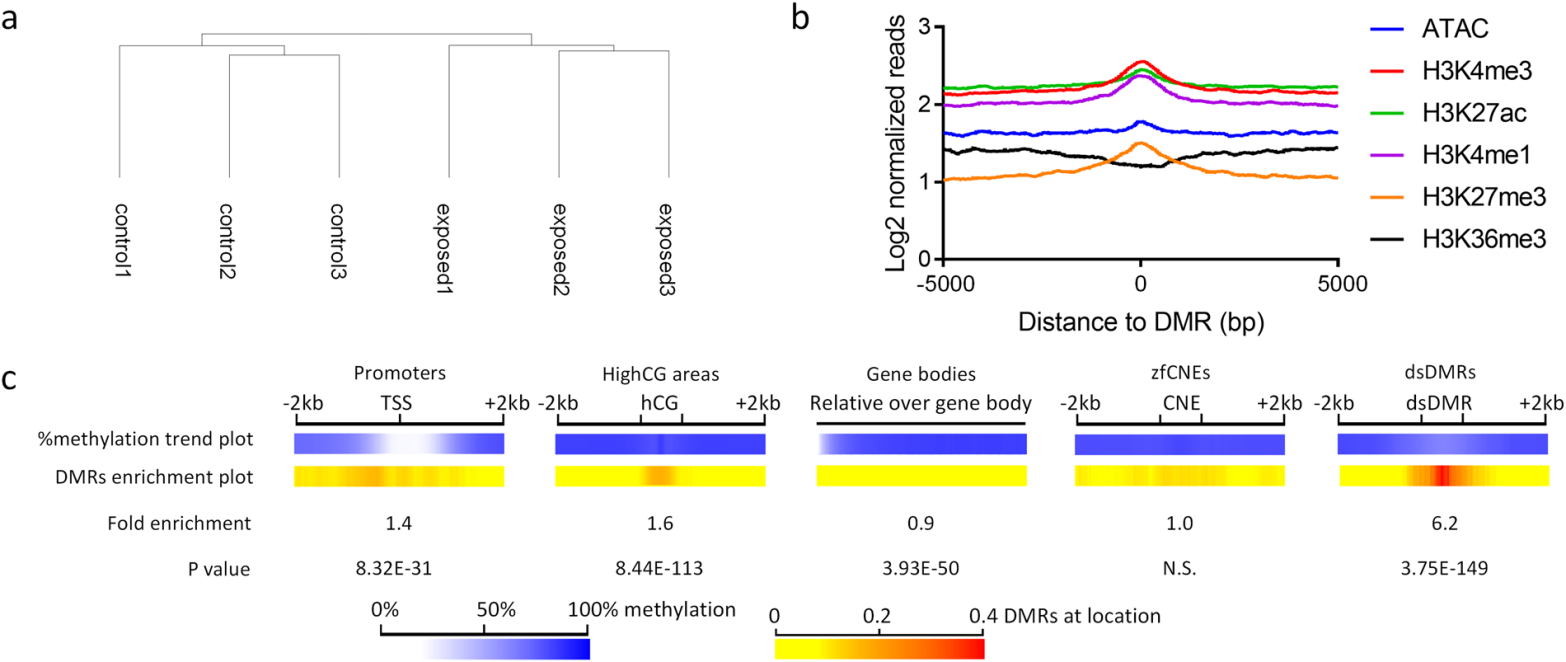
Distribution of DNA methylation and differentially methylation regions (DMRs) over specific features. (**a**) Cluster analysis of all measured CpG sites with at least 5 reads in all replicates (ward clustering). (**b**) Trend plot of different histone post translational modification and ATAC sequencing data surrounding DMRs (**c**) The methylation trend plot shows the percentage methylation over the specific regions, the DMR enrichment plot show the locational preference for DMRs over certain regions. The fold enrichment and P value show the enrichment of DMRs on the specific region over the total amount of measured regions (hypergeometric test). zfCNE: zebrafish non-genic element; dsDMR: developmental stage specific DMR.

We used methylKit to search for differentially methylated regions (DMRs) in F1 zebrafish embryos, using 250 bp tiles with a 125 bp sliding window. Tiles with at least 5 Cs in CpG context containing at least 5 reads per C were selected, allowing sufficient reads for proper statistics. This resulted in 1.6 million tiles that were analysed for differential methylation. Initial cluster analysis showed clear separation of control and exposed samples (Fig. 2a). methylKit analysis revealed 7906 DMRs with a minimum difference of 10%, and no noticeable difference in the number of hyper and hypo methylated regions (3755 vs 4151, respectively) (SI File 1). Merging the nearest (<125 bases) and overlapping DMRs resulted in a final list of 5658 DMRs.

Next, we investigated the regulatory role of DMRs by comparing them to published data sets of accessible chromatin and histone modifications at dome stage. Although this stage deviates from the early gastrula stage in our study (4.3 versus 5.5 hpf), DMRs located at enriched regions of certain histone modifications and/or accessible chromatin might indicate a functional role in gene regulation. Indeed, measuring aligned reads in regions surrounding DMRs revealed clear enrichments in H3K4me3, H3K27me3 and H3K4me1 (Fig. 2b and Supplemental Figure S3). Specifically at regions with relative high intensity of the marks the enrichment is higher near the DMRs (Supplemental Figure S3). H3K4me3 is a modification located at transcriptional start sites of actively transcribed genes, whereas H3K27me3 is often associated with non-expressed genes^62^. Similarly, an enrichment was found at with H3K4me1 (Fig. 2b), a mark that is often associated with developmental enhancers^63^. ATAC and H3K27ac enrichments, which indicate open chromatin and actively transcribed genes^62^ were less pronounced, and a slight negative association was found for H3K36me3, a mark which is co-localized with RNA polymerase II at actively transcribed gene bodies (Fig. 2b).

In order to investigate the relationship between basal methylation at high GC promoters (o/e 0.65, GC content 0.45)^64^ and gene expression, a moderate inverse correlation was observed between the methylation state of a gene promoter and the associated gene expression (Supplemental Figure S4), which is commonly observed by others^65^. The methylation trend over different genomic features exhibited a depletion of methylation at promoter regions for both control and exposed samples (Fig. 2c). We included a list of empirically derived developmental stage specific DMR (dsDMRs) that are dynamically methylated during early zebrafish development^63^, and a computationally derived list of conserved non-genic elements in zebrafish (zfCNEs)^66^. A uniform distribution of generally high levels of CpG methylation was observed at areas with high GC content, zfCNEs and gene bodies, whereas at dsDMRs a slight depletion of DNA methylation was observed (Fig. 2c). At promoter regions, 1144 DMRs were located, which is 1.4x more than what would be expected from a random sampling from all measured tiles (P = 8.32E-31) (Fig. 2c). Interestingly, there is a preference for DMRs upstream of transcriptional start sites (TSS), at the border where hypomethylation starts. Furthermore, a significant number of DMRs were located at high GC areas (1.6x, P = 8.44E-113), and at dsDMRs (6.2x, P = 8.04E-162) (Fig. 2c). No significant overrepresentation of DMRs was found at zfCNEs (Fig. 2c).

### DMRs are non-randomly distributed and moderately associated with differentially expressed genes

Our previous published gene expression dataset^48^ was used to investigate any associations with DMRs. When DMRs were associated to proximal genes (cut-off of ±5 kb from a gene body), 62% of the DMRs were associated to expressed genes, of which 49% were associated to DEGs. We did not observe an inverse correlation between the difference in DMRs (hyper or hypo methylated regions) located at promoters and differentially expressed genes, as was observed in basal methylation levels and gene expression shown in Supplemental Figure S4.

**Figure 3:**
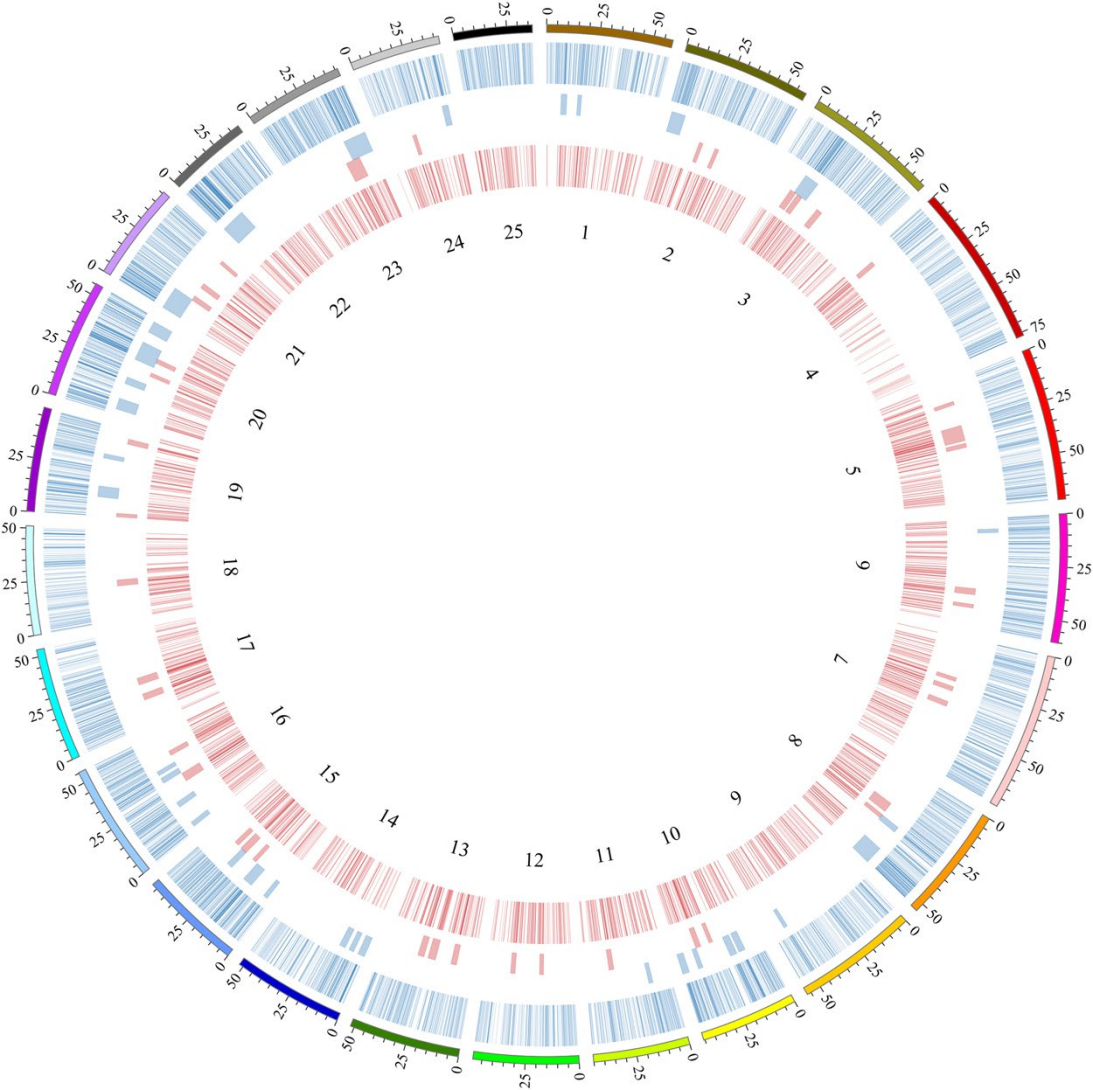
Differential methylation compared to differentially expressed genes. From outside to inside: A circular representation of the zebrafish chromosomes with the number of bases (Mbp), followed by differentially methylated regions (DMRs) with at least 10% difference (blue ring) and blue blocks indicating significant overrepresented clusters of DMRs. Pink blocks indicate overrepresented clusters of differentially expressed genes (DEGs) and the inner pink ring shows the locations of all DEGs (absolute fold change >1.5).

From our DMR list, a non-random pattern of DMRs was visible over the entire genome showing dense regions of DMRs at specific genomic locations (Fig. 3). We calculated overrepresentation of all DMRs over chromosomes and found 33 overrepresented clusters (Fig. 3). When comparing with a background clustering of all measured tiles, some overlap was found (9 out of 33 clusters), indicating that the majority of the 33 clusters were genuine clusters and not a result from non-random distribution of all measured tiles (Supplemental Figure S5). When compiled with a list of differentially expressed genes (DEG, fold change >1.5, FDR < 0.05) derived from the same batch of embryos^48^, a non-random pattern of DEGs was also observed, showing 41 overrepresented gene clusters (Fig. 3). Similar as with the methylation data, comparison with background clustering of all annotated genes revealed some overlapping clusters (13 out of 41; Supplemental Figure S6). Notably, 7 out of 33 DMR clusters overlapped with DEG clusters (Fig. 3).

### Pathway analysis reveals a strong relationship between DEG and DMRs

We used the genomic regions enrichments of annotation tool (GREAT)^56^ to associate genes to DMRs. The resulting gene list was used for KEGG and Ingenuity Pathway Analysis (IPA) to search for enrichments in pathways, upstream regulators and diseases, and compared these with the gene expression dataset. The top 5 KEGG pathways for genes associated to DMRs were metabolic pathways, Wnt signalling, focal adhesion, calcium signalling and MAPK signalling (Table 1).

**Table 1:** KEGG pathway analysis.

Pathway	Genes	adjP
Metabolic Pathways	270	4.56E-25
Wnt signalling	60	5.77E-11
Focal adhesion	67	3.00E-10
Calcium signalling	68	7.05E-10
MAPK signalling	80	7.70E-10

Shown are the enriched pathways, the number of genes involved and the adjusted P value (adjP, hypergeometric test with Benjamini-Hochberg FDR correction).

**Figure 4:**
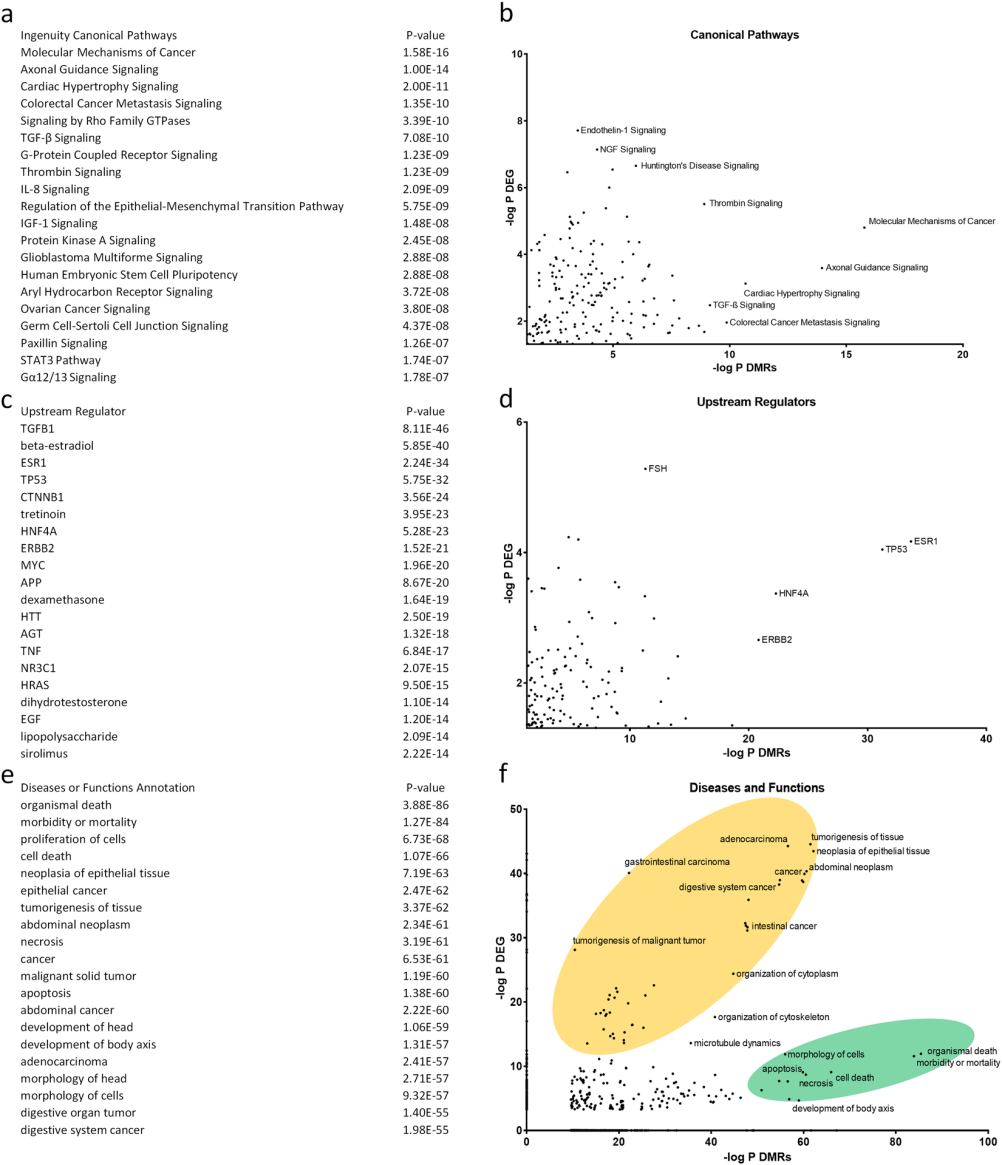
Correlation of overrepresentation between differentially expressed genes (DEGs) and differentially methylated regions (DMRs) following Ingenuity Pathway Analysis. (**a**) Top 20 list of DMR specific enriched canonical pathways. (**b**) Relationship between DEGs and DMRs enriched canonical pathways. (**c**) Top 20 list of DMR specific upstream regulators. (**d**) Relationship between DEGs and DMRs specific enriched upstream regulators. (**e**) Top 20 list of DMR specific diseases and functions. (**f**) Relationship between DEGs and DMRs specific disease lists (cancer: yellow; development and mortality: green).

Following IPA, the top 20 list of DMRs involved in IPAs canonical pathway repository revealed pathways involved in molecular mechanisms of cancer and axonal guidance signalling (Fig. 4a), which were also among the top listed pathways following gene expression analysis (Fig. 4b). Similarly, the top upstream regulators tumour protein 53 (TP53), hepatocyte nuclear factor 4a (HNF4a), and oestrogen receptor 1 (ESR1) (Fig. 4c) were enriched following both gene expression and DNA methylation analysis (Fig. 4d). Diseases and functions related to morbidity and cell death were most prominently enriched with DMRs, but also cancers and developmental disorders like development of body axis and head were among the top 20 diseases or functions (Fig. 4e). Compared to gene expression analysis there is a clear cluster of enriched diseases related to cancers, which has a higher correlation than another cluster which predominantly contains diseases and functions involved in cell death and morbidity (Fig. 4f). In general, IPA analysis of DMRs revealed many similarly enriched pathways, upstream regulators and diseases as compared to DEGs.

Since we found enrichment of DMRs to dsDMRs, we performed IPA analysis on this subset of DMRs, which resulted in the top regulator being FSH, along with similar upstream regulators as compared to all DMRs as TGFB1 and oestrogen related regulators (beta-estradiol and ESR1) (Supplemental Table S3). DMRs specifically located at promoter regions (+/−2 kb) showed enrichments in upstream regulators that more resembled enrichments found at all DMRs, such as TP53, TGFB1, ESR1 and HNF4A (Supplemental Table S3).

### Transcription factor enrichment

**Figure 5:**
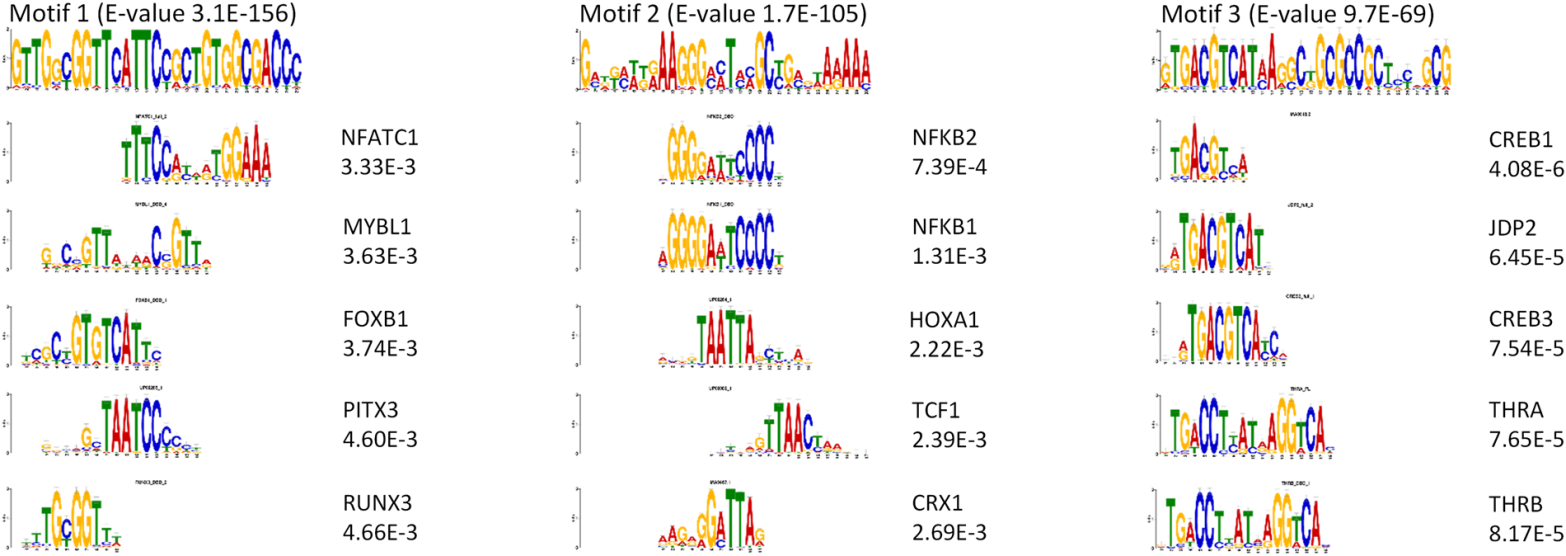
Motif enrichments of differentially methylated regions located near transcriptional start sites. Three most significant motifs as analysed by MEME-ChIP with their respective E-values. Top 5 list of predicted transcription factors from each motif as analysed by Tomtom with their respective P values.

From above it is evident that similar pathways were predicted to be affected at DMRs located near TSSs, as compared to all DMRs. We further noticed that the enrichment of DMRs over the TSS was more pronounced around 500 bases upstream of the TSS (Fig. 2c), suggesting that DMRs upstream of TSSs play an important role in gene regulation via DNA methylation. Therefore, we explored motif enrichment in sequences of DMRs present around TSSs and if these were related to predicted affected pathways obtained from IPA. The three most overrepresented motifs are shown in Fig. 5. Within the first motif, Tomtom analysis revealed similarities with the TFs NFATC, MYBL1, FOXB1, UP00265 and RUNX3 (Fig. 5). The second motif showed similarities with the NFKB1, NFKB2, HOXA1, TCF1 and CRX1 and the third with CREB1, JDP2, CREB3, THRA and THRB. Notably, TFs associated with the third motif were most significant and their genomic locations were almost exclusively (10 out of 12) located at chromosome 4. However, no information is available of the associated genes as the proximate gene functions were generally unknown (Supplemental Table S4).

### Validation of WGBS data

We selected 19 DMRs to validate our WGBS data and to assess transgenerational effects. We based the selection on genes from IPA, together with 2 regions that showed more than 30% difference over a region of more than 1 kb following WGBS analysis (*BX324216.3* and *ostm1*, Supplemental Figure S1). A clear correlation was observed between WGBS data and the BisPCR2 data (Supplemental Figure S7). However, 4 out of the 19 loci (*ccdc25*, *sacs*, *kcnd3* and *epx*) showed no difference in methylation following BisPCR2 analysis (Supplemental Figure S7).

### DNA methylation changes are persistent up to the third generation

**Figure 6:**
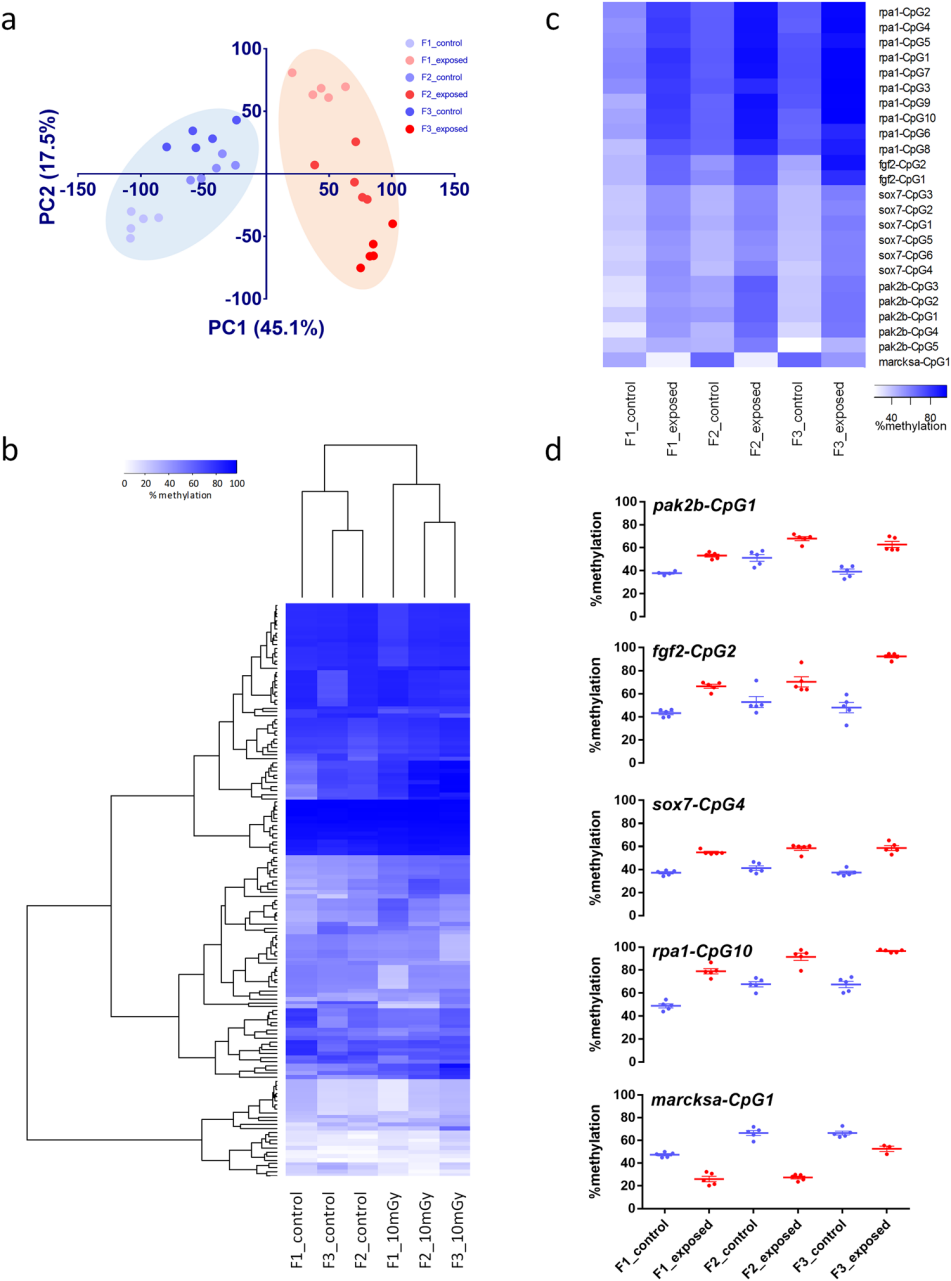
Locus specific analysis of DNA methylation by BisPCR2. (**a**) Principal component analysis of F1, F2 and F3 generations 5.5 hpf embryos from control and 8.7 mGy/h exposed ancestors. (**b**) Hierarchical clustering of all 146 analysed CpG sites and all samples (Ward clustering). (**c**) Heat map of 23 CpG sites that show persistent changes over all generations with at least 10% difference on DNA methylation (P < 0.01). (**d**) Scatterplots of significantly different single CpG sites, showing low variation between replicates and significant persistent changes over generations (P < 0.01, >10% difference).

Our transgenerational assessment of DNA methylation revealed a separation of controls from exposed samples, over all generations following principal component analysis (PCA). Treatments were separated by PC1, which explained 45.1% of the variance, whereas PC2 explained 17.5%, predominantly separating generations within the treatments (Fig. 6a). Additionally, hierarchical clustering analysis separated controls and exposed samples of all generations (Fig. 6b). These results indicate persistent changes of DNA methylation between generations. If we used a 10% cut-off on significant targets over all generations, we found persistent transgenerational effects of DNA methylation at 5 of the 15 true positive targets: myristoylated alanine-rich protein kinase C substrate a (*marcksa*), replication protein A1 (*rpa1*), fibroblast growth factor 2 (*fgf2*), p21 protein (Cdc42/Rac)-activated kinase 2b (*pak2b*), and SRY (sex determining region Y)-box 7 (*sox7*) (Fig. 6c). Generally, these sites showed very low deviation between replicates and showed consistent changes between control and exposed samples over all generations (Fig. 6d).

## Discussion

In this study, we analysed DNA methylation across the entire genome in F1 zebrafish embryos from irradiated parents. Our results revealed large-scale changes in the DNA methylome, which were allocated at certain genomic features, specifically at high content GC areas, promoters and dsDMRs, as well as chromatin marks associated with gene regulation. This implies a regulatory function for these DMRs. When results from gene expression analysis^48^ were compared with the DMRs, a strong association in overrepresented pathways was observed, as assessed by IPA analysis. Interestingly, mutually significant pathways and regulators could be linked back to observed phenotypic effects. Additionally, a selection of DMRs showed persistent changes on DNA methylation up to F3 generation embryos, indicating that changes to the methylome could manifest similar effects in F3 as compared to F1 embryos.

A central dogma in DNA methylation research is the inverse association between promoter methylation at CpG islands and gene expression^11^. Generally, a similar trend was observed within our data, however this association was rather weak and many genes did not follow this relationship, an observation which has been shown before at similar stages of zebrafish^34,35,63^ and mammals^65^.

Since we observed a non-random distribution of DMRs and DEGs, we used an algorithm to scan for overrepresented clusters of DMRs and DEGs over the genome and looked for overlapping regions. First, we compared background clusters of all measured methylation tiles or annotated genes with the DMRs and DEGs, respectively, in order to find out if DMRs and DEGs clusters were not biased by regions covering many methylation tiles or genes. This analysis resulted clusters for both DMRs and DEGs, but overlapped only at 7 locations, indicating limited overlap between gene expression and methylation. Additionally, when comparing promoter DMRs with their respective DEGs, we did not observe any correlation. These results indicate that the inverse association is not present when looking at small changes in DNA methylation. Nevertheless, the resulting 7 overlapping regions might be involved in epigenetic control as previously reported in transgenerational rodent studies, following exposures to environmental contaminants^52^. In that study, overlapping clusters were observed between DEGs and DMRs, indicating the presence of epigenetic control regions involved in transgenerational inheritance induced by exposures to environmental contaminants. However, limited overlap was found when the overlapping background clusters were removed^52^.

Although our results suggest limited involvement of DMRs in proximal differential gene expression, pathway analysis did reveal many similar overrepresented pathways between DNA methylation and gene expression data. In fact, we found many DMRs associated to non-expressed genes and to non-differentially expressed genes. The methylation state associated with non-expressed or non-differentially expressed genes could be an indication of a poised status of the respective promoter, and collectively with changes on histone marks (e.g. H3K4me3, H3K9me3 and H3K27me3)^64,67^, these genes could be expressed at later developmental stages. We therefore compared our results with published data sets of different chromatin marks. Although this comparison was performed between two different developmental stages (dome vs 50% epiboly), enrichments of such chromatin marks around DMRs might indicate regulatory functions. Indeed, our enrichment analysis of DMRs at specific chromatin marks, such as H3K4me3, H3K4me1 and H3K27ac/me3, indicated the involvement of DMRs at such genomic regions, whereas H3K36me3 showed a slight depletion around DMRs. Also, at early gastrula stage most maternal transcripts are degraded and the zygotic genome is activated, but many genes involved in later stages of development are not yet expressed^68^, which could indicate changes of DNA methylation at genes poised for transcription.

We hypothesized that DMRs should be located at regulatory regions and be linked to specific gene pathways. Indeed, when we used GREAT to associate DMRs to genes, KEGG and IPA revealed many predicted affected pathways, and interestingly many of those pathways could be linked to observed effects at higher biological levels. The most significant pathway derived from IPA, molecular mechanisms of cancer, was accompanied with many cancer related disease pathways, which could be expected following ionizing radiation exposure. However, in this study we assessed the progeny of exposed parents, indicating that observed DMRs are related to parentally affected genes. We were able to compare DNA methylation results with gene expression, and observed similarity in overrepresented pathways and regulators. Tumour protein 53 (TP53), hepatocyte nuclear factor 4 alpha (HNF4a) and transforming growth factor beta 1 (TGFB1) were overrepresented in both DMRs and DEG analyses. These three regulators are involved as suppressors in tumorigenesis^69–71^, where TP53 is thought to play a central role in ionizing radiation response in zebrafish^69^. Interestingly, the involvement of TP53, can be linked to the increase in DNA damage in the offspring of exposed parents, found in our parallel study^47^. In the same study possible inflammation in the embryos was also reported, which corroborates with the involvement of many regulators and pathways involved in inflammatory response (e.g. TGFB1, IL8, TNF, STAT3). Other overrepresented pathways following gene expression as well as DNA methylation analysis could indicate effects that manifest later in life, such as axonal guidance signalling involved in neurodevelopment, and FSH signalling linked to reproductive outcome. Interestingly, irradiated parents exhibited effects on ovaries with an increase in pre-vitellogenic follicles, indicating effects on maturation of oocytes, which could involve FSH signalling^50^. These results indicate potential novel affected molecular mechanisms in radiation response.

Compared to previous studies involving ionizing radiation and genome wide DNA methylation analysis, the KEGG analysis showed similar overrepresented pathways. For example, in whole blood from mice exposed to 0.5 Gy X-rays, metabolic pathways, focal adhesion and development of body axis were overrepresented^28^. Furthermore, DNA methylation changes were observed in an irradiated breast cancer cell line exposed to 2 and 6 Gy following exposure to X-rays. Here, apoptosis pathways and processes involved in cell cycle were affected^27^, similar pathways that we observed in our data. Notably, these studies were performed on acutely exposed mice and cell lines, whereas our results were observed in progeny from irradiated parents, and indicates that different genotoxic exposure scenarios may affect similar pathways.

Our data show that similar pathways were predicted to be affected at DMRs located near TSSs, as compared to all DMRs. We further noticed that the enrichment of DMRs over the TSS was more pronounced around 500 bases upstream of the TSS, which indicates that DMRs upstream of TSSs play an important role in gene regulation via DNA methylation. When searching for overrepresented motifs in these regions a number of predicted transcription factors were related to cancers and oxidative stress, like nuclear factor kappa beta (NFKB1 and 2), jun dimerization protein 2 (JDP2), myb proto-oncogene like 1 (MYBL1) and runt-related transcription factor 3 (RUNX3). Interestingly, these TFs can be related back to the overrepresented disease functions related to cancer. Collectively, the similarity in pathways between DNA methylation and gene expression together with our phenotypic assessment, exemplifies the functional role of DNA methylation in zebrafish.

The effects on DNA methylation could be linked back to phenotypes, possibly by changes in the germline of the parental lines. However, in our set-up we used zebrafish that were irradiated one year before. The results obtained here are therefore most likely a combination of the initial insult and the accumulated effects post-exposure over one year. This limits us in our conclusions whether the effects are genuine effects from irradiation or an indirect effect thereof. Furthermore, this limits us in addressing whether the changes in DNA methylation results from the insult, or vice versa. Nevertheless, the DMRs have been (a) induced as a consequence of the exposure and (b) were partly conserved in F2 and F3.

We selected 19 DMRs to validate our results and to assess transgenerational effects. In our initial analysis we used a 10% cut-off as a relevant effect size. This difference might have a significant effect on phenotype, but a proportion of DMRs might have no effect. In general, 10% difference or lower is often used as a cut-off for such analysis^45,72^, however, mechanistic studies are needed to address this issue^61,73^. In order to get an estimate of the type II error rate we performed a validation of the targets by selecting 18 DMRs that were included in the IPA analysis as well as two DMRs that exhibited differential methylation of more than 30% over a region of more than 1 kb. In general, we found a strong correlation between WGBS data and BisPCR2 analysis, however 4 targets did not show any difference on DNA methylation in the validation set. One shortcoming of using WGBS is the generally low read depth of sequences, which is a consequence of measuring the entire genome for changes of DNA methylation at a certain depth, while keeping the costs manageable. Each read represents one clone of genomic DNA, which is derived from one cell of one embryo out of a pool of 100 embryos, each consisting of approximately 8,000 cells^74,75^. The chance is present that within three replicate measurements the reads at one locus represent different cell types between exposures, resulting in an observed change in methylation. Therefore, depending on the number of replicates and sequencing depth, there is a chance of false positives, which cannot be detected by any statistical method. Although we attempted to minimize bias in cell type by using early gastrula stage embryos, we observed 4 false positives out of 19 analysed targets, which indicated that approximately 80% of the DMRs are true differentially methylated regions. This exemplifies the need for proper validation in genome wide methylation studies.

Another constraint in our study is the fact that the founder generation is derived from a one-tank experimental set-up, which did not allow assessing aquarium to aquarium differences. We aimed to minimize these differences by keeping all aquaria in the same system. Additionally, the transgenerational study involved control tanks from F1, F2 and F3. Even though these controls were from different generations, our cluster analysis showed that these controls were more related to each other than to the exposed populations, where the exposures explained 45.1% of the variation and the generations were more separated by the 2^nd^ PC (17.5%). Although variation in methylation is also apparent within the controls and exposed samples between generations, this data suggest that the observed effects on DNA methylation were predominantly accountable to the exposure effects. However, we cannot entirely exclude that differences were attributed to different genetic backgrounds, although our gene expression data did not show any changes of global mutation rates between F1 exposed and control samples^48^. Therefore, it is more likely that the variation within generations is explained by the way F1, F2 and F3 were produced.

We found persistent transgenerational effects on DNA methylation in 5 of the 15 true positive targets: myristoylated alanine-rich protein kinase C substrate a (*marcksa*), replication protein A1 (*rpa1*), fibroblast growth factor 2 (*fgf2*), p21 protein (Cdc42/Rac)-activated kinase 2b (*pak2b*), and SRY (sex determining region Y)-box 7 (*sox7*). *Marcksa* is involved in zebrafish development via actin cytoskeleton homeostasis^76^. Phenotypic effects of 53 mGy/h exposed parents’ progeny showed disintegration of cell structure and complete growth arrest around 8hpf, implying an effect on actin cytoskeleton^47^. Similar to *marcksa*, the Pak protein family is also involved in cytoskeleton function^77^. Interestingly, PAK2 has been shown to be activated following ionizing radiation exposure in mammalian cell lines^78^. Persistent changes were also found at a region behind the gene coding region of *rpa1*, a single-stranded DNA binding protein, involved in DNA replication and repair^79^. Both *fgf2* and *sox7* are involved in angiogenesis^80^ and cardio vascular development^81^. Together, these results suggest persistent changes on a subset of DMRs, which could cause adverse effects over generations. Such DMRs could be used as biomarkers for monitoring methylation changes in ecotoxicological transgenerational studies to radiation.

To conclude, we have analysed DNA methylation in progeny from gamma irradiated F0 zebrafish, resulting in a vast number of differentially methylated regions, of which many could be associated to pathways involved in cancers and apoptosis. Our transgenerational assessment revealed over 30% of the analysed loci persistently changed over 3 generations. Since DMRs can be linked to function, transgenerational effects can result in aberrant phenotypes, possibly affecting development. Although, these results aid in our understanding of how phenotypes persist over generations, more mechanistic studies are warranted (e.g. locus specific epigenome editing^82^) to gain insight in the causation of such effects and to directly link functional effects to DNA methylation. Ultimately, such studies will provide the knowledge whether DMRs could be used as biomarkers for monitoring methylation changes in ecotoxicological transgenerational studies to radiation.

## Electronic supplementary material

Supplementary Information

Supplementary Information file 1

## References

[1] Unscear. *Effects of ionizing radiation: UNSCEAR2006 Report to the General Assembly, with scientific annexes*. 2, (United Nations Publications, 2009).

[2] Foley TP, Límanová Z, Potluková E (2015). Medical consequences of Chernobyl with focus on the endocrine system: Part 1. Cas. Lek. Cesk..

[3] Foley TP, Límanová Z, Potluková E (2015). Medical Consequences of Chernobyl with Focus on the Endocrine System - Part 2. Cas. Lek. Cesk..

[4] Merrifield M, Kovalchuk O (2013). Epigenetics in radiation biology: a new research frontier. Front. Genet..

[5] Barber RC, Dubrova YE (2006). The offspring of irradiated parents, are they stable?. Mutat. Res. Mol. Mech. Mutagen..

[6] Jirtle RL, Skinner MK (2007). Environmental epigenomics and disease susceptibility. Nat. Rev. Genet..

[7] Godfrey KM, Lillycrop KA, Burdge GC, Gluckman PD, Hanson MA (2007). Epigenetic mechanisms and the mismatch concept of the developmental origins of health and disease. Pediatr. Res..

[8] Hanson MA, Skinner MK (2016). Developmental origins of epigenetic transgenerational inheritance. Environ. epigenetics.

[9] Miska EA, Ferguson-smith AC (2016). Transgenerational inheritance: Models and mechanisms of non – DNA sequence – based inheritance. Science.

[10] Wang Yan, Liu Huijie, Sun Zhongsheng (2017). Lamarck rises from his grave: parental environment-induced epigenetic inheritance in model organisms and humans. Biological Reviews.

[11] Jones Pa (2012). Functions of DNA methylation: islands, start sites, gene bodies and beyond. Nat. Rev. Genet..

[12] Tillo D, Mukherjee S, Vinson C (2016). Inheritance of Cytosine Methylation. J. Cell. Physiol..

[13] Chamorro-Garcia, R. *et al*. Ancestral perinatal obesogen exposure results in a transgenerational thrifty phenotype in mice. *Nat. Commun*. **8**, (2017). 10.1038/s41467-017-01944-z PMC5722856 29222412

[14] Dolinoy DC, Huang D, Jirtle RL (2007). Maternal nutrient supplementation counteracts bisphenol A-induced DNA hypomethylation in early development. Proc. Natl. Acad. Sci. USA.

[15] Olsvik PA (2014). Impacts of TCDD and MeHg on DNA methylation in zebrafish (Danio rerio) across two generations. Comp. Biochem. Physiol. Part C Toxicol. Pharmacol..

[16] Kamstra JH, Sales LB, Aleström P, Legler J (2017). Differential DNA methylation at conserved non-genic elements and evidence for transgenerational inheritance following developmental exposure to mono(2-ethylhexyl) phthalate and 5-azacytidine in zebrafish. Epigenetics Chromatin.

[17] Carvan MJ (2017). Mercury-induced epigenetic transgenerational inheritance of abnormal neurobehavior is correlated with sperm epimutations in zebrafish. PLoS One.

[18] Trijau Marie, Asselman Jana, Armant Olivier, Adam-Guillermin Christelle, De Schamphelaere Karel A. C., Alonzo Frédéric (2018). Transgenerational DNA Methylation Changes in Daphnia magna Exposed to Chronic γ Irradiation. Environmental Science & Technology.

[19] Pogribny I, Raiche J, Slovack M, Kovalchuk O (2004). Dose-dependence, sex- and tissue-specificity, and persistence of radiation-induced genomic DNA methylation changes. Biochem. Biophys. Res. Commun..

[20] Kovalchuk O (2004). Methylation changes in muscle and liver tissues of male and female mice exposed to acute and chronic low-dose X-ray-irradiation. Mutat. Res..

[21] Kalinich JF, Catravas GN, Snyder SL (1989). The effect of gamma radiation on DNA methylation. Radiat. Res..

[22] Loree J (2006). Radiation-induced molecular changes in rat mammary tissue: possible implications for radiation-induced carcinogenesis. Int. J. Radiat. Biol..

[23] Kutanzi K, Kovalchuk O (2013). Exposure to estrogen and ionizing radiation causes epigenetic dysregulation, activation of mitogen-activated protein kinase pathways, and genome instability in the mammary gland of ACI rats. Cancer Biol. Ther..

[24] Koturbash I (2016). Radiation-induced changes in DNA methylation of repetitive elements in the mouse heart. Mutat. Res..

[25] Prior S (2016). Densely ionizing radiation affects DNA methylation of selective LINE-1 elements. Environ. Res..

[26] Newman MR (2014). The Methylation of DNA Repeat Elements is Sex-Dependent and Temporally Different in Response to X Radiation in Radiosensitive and Radioresistant Mouse Strains. Radiat. Res..

[27] Antwih DA, Gabbara KM, Lancaster WD, Ruden DM, Zielske SP (2013). Radiation-induced epigenetic DNA methylation modification of radiation-response pathways. Epigenetics.

[28] Wang Jingzi, Zhang Youwei, Xu Kai, Mao Xiaobei, Xue Lijun, Liu Xiaobei, Yu Hongjun, Chen Longbang, Chu Xiaoyuan (2014). Genome-Wide Screen of DNA Methylation Changes Induced by Low Dose X-Ray Radiation in Mice. PLoS ONE.

[29] Song W (2014). Increased P16 DNA methylation in mouse thymic lymphoma induced by irradiation. PLoS One.

[30] Tamminga J (2008). Paternal cranial irradiation induces distant bystander DNA damage in the germline and leads to epigenetic alterations in the offspring. Cell Cycle.

[31] Koturbash I (2006). Epigenetic dysregulation underlies radiation-induced transgenerational genome instability *in vivo*. Int. J. Radiat. Oncol..

[32] Choi VWY, Yu KN (2015). Embryos of the zebrafish Danio rerio in studies of non-targeted effects of ionizing radiation. Cancer Lett..

[33] Kamstra JH, Aleström P, Kooter JM, Legler J (2015). Zebrafish as a model to study the role of DNA methylation in environmental toxicology. Environ. Sci. Pollut. Res..

[34] Potok ME, Nix Da, Parnell TJ, Cairns BR (2013). Reprogramming the maternal zebrafish genome after fertilization to match the paternal methylation pattern. Cell.

[35] Jiang L (2013). Sperm, but not oocyte, DNA methylome is inherited by zebrafish early embryos. Cell.

[36] Wu H, Zhang Y (2014). Reversing DNA Methylation: Mechanisms, Genomics, and Biological Functions. Cell.

[37] Smith ZD (2012). A unique regulatory phase of DNA methylation in the early mammalian embryo. Nature.

[38] Kamstra JH, Løken M, Aleström P, Legler J (2015). Dynamics of DNA hydroxymethylation in zebrafish. Zebrafish.

[39] Strömqvist M, Tooke N, Brunström B (2010). DNA methylation levels in the 5′ flanking region of the vitellogenin I gene in liver and brain of adult zebrafish (Danio rerio)—Sex and tissue differences and effects of 17α-ethinylestradiol exposure. Aquat. Toxicol..

[40] Li D (2009). Developmental mechanisms of arsenite toxicity in zebrafish (Danio rerio) embryos. Aquat. Toxicol..

[41] Fang X, Thornton C, Scheffler BE, Willett KL (2013). Benzo[a]pyrene decreases global and gene specific DNA methylation during zebrafish development. Environ. Toxicol. Pharmacol..

[42] Corrales J (2014). Effects on specific promoter DNA methylation in zebrafish embryos and larvae following benzo[a]pyrene exposure. Comp. Biochem. Physiol. Part - C Toxicol. Pharmacol..

[43] Aluru N (2015). Developmental exposure to 2, 3, 7, 8-tetrachlorodibenzo-p-dioxin alters DNA methyltransferase (dnmt) expression in zebrafish (Danio rerio). Toxicol. Appl. Pharmacol..

[44] Ceccaldi A (2011). C5-DNA methyltransferase inhibitors: from screening to effects on zebrafish embryo development. Chembiochem.

[45] Bouwmeester MC (2016). Zebrafish embryos as a screen for DNA methylation modifications after compound exposure. Toxicol. Appl. Pharmacol..

[46] Kong E, Cheng S, Yu K (2016). Zebrafish as an *In Vivo* Model to Assess Epigenetic Effects of Ionizing Radiation. Int. J. Mol. Sci..

[47] Hurem S (2017). Parental gamma irradiation induces reprotoxic effects accompanied by genomic instability in zebrafish (Danio rerio) embryos. Environ. Res..

[48] Hurem S (2018). Parental exposure to gamma radiation causes progressively altered transcriptomes linked to adverse effects in zebrafish offspring. Environ. Pollut..

[49] Hinton TG (2007). Radiation-induced effects on plants and animals: findings of the United Nations Chernobyl Forum. Health Phys..

[50] Hurem S (2018). Gamma irradiation during gametogenesis in young adult zebrafish causes persistent genotoxicity and adverse reproductive effects. Ecotoxicol. Environ. Saf..

[51] Akalin A (2012). methylKit: a comprehensive R package for the analysis of genome-wide DNA methylation profiles. Genome Biol..

[52] Haque MM, Nilsson EE, Holder LB, Skinner MK (2016). Genomic Clustering of differential DNA methylated regions (epimutations) associated with the epigenetic transgenerational inheritance of disease and phenotypic variation. BMC Genomics.

[53] Chen Y (2017). Using local chromatin structure to improve CRISPR/Cas9 efficiency in zebrafish. PLoS One.

[54] Bogdanovic O (2012). Dynamics of enhancer chromatin signatures mark the transition from pluripotency to cell specification during embryogenesis. Genome Res..

[55] Zhang Y (2014). Canonical nucleosome organization at promoters forms during genome activation. Genome Res..

[56] McLean CY (2010). GREAT improves functional interpretation of cis-regulatory regions. Nat. Biotechnol..

[57] Machanick P, Bailey TL (2011). MEME-ChIP: Motif analysis of large DNA datasets. Bioinformatics.

[58] Gupta S, Stamatoyannopoulos JA, Bailey TL, Noble WS (2007). Quantifying similarity between motifs. Genome Biol..

[59] Bernstein DL, Kameswaran V, Le Lay JE, Sheaffer KL, Kaestner KH (2015). The BisPCR2 method for targeted bisulfite sequencing. Epigenetics Chromatin.

[60] Wagner DE (2018). Single-cell mapping of gene expression landscapes and lineage in the zebrafish embryo. Science (80-.)..

[61] Greally, J. M. & Jacobs, M. N. *In Vitro* and *In Vivo* Testing Methods of Epigenomic Endpoints for Evaluating Endocrine Disruptors. 445–471 (2013). 10.14573/altex.2013.4.445 24173168

[62] Bannister AJ, Kouzarides T (2011). Regulation of chromatin by histone modifications. Cell Res..

[63] Lee HJ (2015). Developmental enhancers revealed by extensive DNA methylome maps of zebrafish early embryos. Nat. Commun..

[64] Andersen IS, Reiner AH, Aanes H, Aleström P, Collas P (2012). Developmental features of DNA methylation during activation of the embryonic zebrafish genome. Genome Biol..

[65] Bock C (2012). Analysing and interpreting DNA methylation data. Nat. Rev. Genet..

[66] Hiller M (2013). Computational methods to detect conserved non-genic elements in phylogenetically isolated genomes: Application to zebrafish. Nucleic Acids Res..

[67] Du J, Johnson LM, Jacobsen SE, Patel DJ (2015). DNA methylation pathways and their crosstalk with histone methylation. Nat. Rev. Mol. Cell Biol..

[68] Aanes H (2011). Zebrafish mRNA sequencing deciphers novelties in transcriptome dynamics during maternal to zygotic transition. Genome Res..

[69] Guo L (2012). Ionizing radiation induces a dramatic persistence of p53 protein accumulation and DNA damage signaling in mutant p53 zebrafish. Oncogene.

[70] Walesky C, Apte U (2015). Role of hepatocyte nuclear factor 4alpha (HNF4alpha) in cell proliferation and cancer. Gene Expr.

[71] Principe DR (2014). TGF-β: duality of function between tumor prevention and carcinogenesis. J. Natl. Cancer Inst..

[72] Martinez-Arguelles DB, Papadopoulos V (2015). Identification of Hot Spots of DNA Methylation in the Adult Male Adrenal in Response to In Utero Exposure to the Ubiquitous Endocrine Disruptor Plasticizer Di-(2-ethylhexyl) Phthalate. Endocrinology.

[73] Marczylo EL, Jacobs MN, Gant TW (2016). Environmentally induced epigenetic toxicity: potential public health concerns. Crit. Rev. Toxicol..

[74] Kobitski AY (2015). An ensemble-averaged, cell density-based digital model of zebrafish embryo development derived from light-sheet microscopy data with single-cell resolution. Sci. Rep..

[75] Kane DA, Kimmel CB (1993). The zebrafish midblastula transition. Development.

[76] Ott LE (2011). Two Myristoylated Alanine-Rich C-Kinase Substrate (MARCKS) Paralogs are Required for Normal Development inZebrafish. Anat. Rec. Adv. Integr. Anat. Evol. Biol..

[77] Hofmann C, Shepelev M, Chernoff J (2004). The genetics of Pak. J. Cell Sci..

[78] Roig J, Traugh JA (1999). p21-activated Protein Kinase -PAK Is Activated by Ionizing Radiation and Other DNA-damaging Agents: SIMILARITIES AND DIFFERENCES TO -PAK. J. Biol. Chem..

[79] Waga S, Stillman B (1998). The DNA replication fork in eukaryotic cells. Annu. Rev. Biochem..

[80] Nicoli S, De Sena G, Presta M (2009). Fibroblast growth factor 2-induced angiogenesis in zebrafish: The zebrafish yolk membrane (ZFYM) angiogenesis assay. J. Cell. Mol. Med..

[81] Wat JJ, Wat MJ (2014). Sox7 in vascular development: Review, insights and potential mechanisms. Int. J. Dev. Biol..

[82] Lei Y (2017). Targeted DNA methylation *in vivo* using an engineered dCas9-MQ1 fusion protein. Nat. Commun..

