# Supplementary material for: Ionizing radiation induces transgenerational effects of DNA methylation in zebrafish
Source: Sci Rep. 2018 Oct 18;8:15373. doi: 10.1038/s41598-018-33817-w (PMC6193964; doi:10.1038/s41598-018-33817-w)
Supplement: Supplementary file 1 — Supplementary Information [file 41598_2018_33817_MOESM1_ESM.docx]

Supporting Information

Ionizing radiation induces transgenerational effects of DNA methylation in zebrafish.

Jorke H Kamstra^1*^, Selma Hurem^1^, Leonardo Martin Martin^1 2^, Leif C Lindeman^1 3^, Juliette Legler^45^, Deborah Oughton^3^, Brit Salbu^3^, Dag Anders Brede^3^, Jan Ludvig Lyche^1^, Peter Aleström^1^

^1^Faculty of Veterinary Medicine and Biosciences, Dept. of Basic Science and Aquatic Medicine, CoE CERAD, Norwegian University of Life Sciences, 0033 Oslo, Norway.

^2^University of Camagüey, Faculty of Agropecuary Sciences, Camagüey 70100, Cuba.

^3^Faculty of Environmental Sciences and Natural Resource Management, CERAD CoE, Norwegian University of Life Sciences, 1433 Ås, Norway.

^4^Institute for Environment, Health and Societies, College of Health and Life Sciences, Brunel University London, Uxbridge, United Kingdom, [juliette.legler@brunel.ac.uk](mailto:juliette.legler@brunel.ac.uk)

^5^Utrecht University, Institute for Risk Assessment Sciences, P.O. Box 80177, NL-3508 TD Utrecht, The Netherlands

**Supplemental materials and methods**

Husbandry housing parameters

Six month old males and females (F0) (N = 30) were kept at 28 ± 1°C on a 14 - 10 hour light-dark cycle at a density of up to 10 fish/L. The system water (SW) was prepared from particle and active charcoal filtrated reverse osmosis deionized tap water, which was kept sterile by UV irradiation. SW was conditioned by adding 155 mg synthetic sea salt (Instant Ocean, Blacksburg, USA), 53 mg sodium carbonate and 15 mg calcium chloride (Sigma-Aldrich, Norway AS) per liter to reach a conductivity of 500 µS/cm, a general hardness (GH) of 4 – 5, and a pH 7.5 (adjusted with 1M HCl). Adults were fed with Gemma Micro 300 (Skretting, Stavanger, Norway) dry feed twice a day and artemia (Scanbur, Copenhagen, Denmark) once a day. Health monitoring was performed by daily inspection. Sentinels were sent to ZIRC for pathology and were assessed for microbiological status in 6 months intervals. The NMBU zebrafish facility and SOPs has AAALAC accreditation (No. 1036) and is approved by the National Animal Research Authority. All experiments were performed according to Norwegian Animal Welfare Act (2009) and the EU Directive 2010/63.

*DNA purification*

DNA was purified with the Puregene tissue DNA extraction kit (Qiagen, Germany), with modifications. Embryos were lysed in 300 µL lysis buffer and disrupted with the use of a 20G needle. To remove RNA, 5 µL of 20 mg/mL RNAse A (Qiagen, Germany) was added, followed by an overnight proteinase K incubation step (20 µL of 20 mg/mL) (Qiagen, Germany) at 65 °C, under continuous shaking at 300 rpm. Following incubation, an extra 5 µL RNAse A was added and left at room temperature for 1 hr to ensure complete degradation of RNAs. Protein precipitation buffer (supplied by the DNA extraction kit) was added (100 µL) and after vigorously shaking, the precipitate was centrifuged at 13,000 rpm for 3 min. Supernatant was added to a 1.5 mL reaction tube containing 300 µL isopropanol, supplemented with 90 µL of 3M sodium acetate (pH 5.3), and was mixed by inverting 50 times and centrifuged for 1 min at 13,000 rpm. Supernatant was discarded and the pellet was rinsed twice with 70 % freshly prepared ethanol (300 µL) by gentle shaking and centrifugation at 13,000 rpm for 1 min. The pellet was air dried for 5 minutes and dissolved in hydration buffer, supplied with the kit. DNA was assessed for RNA contamination and length with gel electrophoreses and measured for concentration and quality by Nanodrop (ND-1000; Thermo Scientific). DNA was stored at -20 °C until further analysis.

*Whole genome bisulfite sequencing - library prep*

Library preparation and sequencing was performed at Novogene (Hong Kong). DNA was sheared into 200-300 bp fragments using the Covaris system (S220, Covaris, US). Library was prepared using the Illumina TruSeq DNA methylation kit (Illumina, US). DNA was terminal repaired, A-ligated, followed by ligation of methylated adapters according to the Illumina tru-seq protocol. Each library was bisulfite converted using the EZ DNA methylation gold kit (Zymo Research, US). Subsequently, the library was size selected and PCR amplified. Library was checked for quality and average size by Agilent bioanalyzer 2100, and concentration was determined by qPCR. Each library was analyzed on a separate lane on the HiSeq 4000 (Illumina, USA) in paired end mode using 150 bp sequence length.

*BisPCR2*

We used the BisPCR2 method^1^, which was adapted at our lab and is extensively described^2^. In short, the method uses specific primers designed for bisulfite converted DNA (Epitect kit, Qiagen, Germany), with an Illumina adapter overhang (SI Table S1). Primers were developed using the online Bisearch tool (<http://bisearch.enzim.hu/>), and validated for specificity and amplicon size by gel electrophoresis, as described previously^2^. From every bisulfite converted DNA sample, PCRs for each of the 20 targets were performed, these were pooled in equal volumes, purified (PCR purification kit, Qiagen, Germany) and subjected to a second PCR with Illumina specific barcoded primers (sequences described previously by Bernstein et al. (2015)). Following the second PCR, products were purified with AMPure XP beads (Beckman Coulter, USA), and measured using Qubit. Random samples were analysed on the Agilent 2100 bioanalyzer for library quality, which showed high quality libraries (data not shown). Concentrations were converted to nM with c (nM) = (Qubit conc (ng/µL) * 1,000,000) / (660 * average amplicon length). All second PCR products were pooled in equimolar portions to a final concentration of 2 nM, followed by 150 base paired end sequencing analysis on MiSeq (Illumina, USA) as described previously^1^.

Table S1: Primer sequences for BisPCR2 analysis. Table shows primers sequences per target, with the expected amplicon length for sequencing and the optimized annealing temperature for the PCR reaction (Ann. Temp).

| no | name | Sequence | length | Ann. Temp |
| --- | --- | --- | --- | --- |
| 1 | fbxo46-F | ACACTCTTTCCCTACACGACGCTCTTCCGATCT**GTTATTTTATAGATTTTTAGTTT** | 517 | 52 |
|  | fbxo46-R | GTGACTGGAGTTCAGACGTGTGCTCTTCCGATCT**AAACCATTACATACACACTT** |  |  |
| 2 | myo1c-F | ACACTCTTTCCCTACACGACGCTCTTCCGATCT**TTTTGTTTGATTGTGAGTGT** | 439 | 52 |
|  | myo1c-F | GTGACTGGAGTTCAGACGTGTGCTCTTCCGATCT**AACTATTCCTTTAATATACCTCC** |  |  |
| 3 | sacs-F | ACACTCTTTCCCTACACGACGCTCTTCCGATCT**AATTATTGAAAAAAGGTAAGG** | 440 | 52 |
|  | sacs-R | GTGACTGGAGTTCAGACGTGTGCTCTTCCGATCT**AACAACTACACATTTAAAAATAA** |  |  |
| 4 | ccdc25-F | ACACTCTTTCCCTACACGACGCTCTTCCGATCT**AAGATTTATTTGGGGTATTTG** | 397 | 52 |
|  | ccdc25-R | GTGACTGGAGTTCAGACGTGTGCTCTTCCGATCT**CCCTATCACCTTTCTTATAAATA** |  |  |
| 5 | atm-F | ACACTCTTTCCCTACACGACGCTCTTCCGATCT**AATGTTATAGATTATTTTTAGGG** | 497 | 54 |
|  | atm-R | GTGACTGGAGTTCAGACGTGTGCTCTTCCGATCT**CAATTCATAAAATCACACACAAC** |  |  |
| 6 | epx-F | ACACTCTTTCCCTACACGACGCTCTTCCGATCT**GATTTTTTAAGTTGAGGGTT** | 419 | 52 |
|  | epx-R | GTGACTGGAGTTCAGACGTGTGCTCTTCCGATCT**TCCCTATCCTAAAATCAATAAA** |  |  |
| 7 | marcksa-F | ACACTCTTTCCCTACACGACGCTCTTCCGATCT**GTAGTTTTTATGATGTAAGATGAG** | 480 | 52 |
|  | marcksa-R | GTGACTGGAGTTCAGACGTGTGCTCTTCCGATCT**ACTATCCCAACATATCAAAAAA** |  |  |
| 8 | tpd52-F | ACACTCTTTCCCTACACGACGCTCTTCCGATCT**GATAGTATTTGTTTTAAAGTTATT** | 415 | 52 |
|  | tpd52-R | GTGACTGGAGTTCAGACGTGTGCTCTTCCGATCT**CAACTCTTATTTTACACACTCA** |  |  |
| 9 | pak2b-F | ACACTCTTTCCCTACACGACGCTCTTCCGATCT**TGTTAGATGAAGAGTATAGATAAA** | 329 | 54 |
|  | pak2b-R | GTGACTGGAGTTCAGACGTGTGCTCTTCCGATCT**AACATAAATCCAAATAAACTCC** |  |  |
| 10 | crk-F | ACACTCTTTCCCTACACGACGCTCTTCCGATCT**ATTATGTTTGTTTGTTTAGGT** | 393 | 52 |
|  | crk-R | GTGACTGGAGTTCAGACGTGTGCTCTTCCGATCT**TTCACTCTTTTTTTAACACC** |  |  |
| 11 | sox7-F | ACACTCTTTCCCTACACGACGCTCTTCCGATCT**GTTTGTTTATGTGTTATAAGTGA** | 314 | 52 |
|  | sox7-R | GTGACTGGAGTTCAGACGTGTGCTCTTCCGATCT**ATCTCATTCCTTCTTAAACCAA** |  |  |
| 12 | sod3a-F | ACACTCTTTCCCTACACGACGCTCTTCCGATCT**GTTTTTTTTAAAGTGTTAGATAG** | 314 | 52 |
|  | sod3a-R | GTGACTGGAGTTCAGACGTGTGCTCTTCCGATCT**CCTTTTATACCCTTATTTATATTCC** |  |  |
| 13 | fgf2-F | ACACTCTTTCCCTACACGACGCTCTTCCGATCT**TGATAAATAAAGGGATTATAAGT** | 389 | 52 |
|  | fgf2-R | GTGACTGGAGTTCAGACGTGTGCTCTTCCGATCT**CTCAAATAAAAACTAACTCACA** |  |  |
| 14 | kcnd3-F | ACACTCTTTCCCTACACGACGCTCTTCCGATCT**GAAGGTTAAGGATGTTTAATTAG** | 368 | 52 |
|  | kcnd3-R | GTGACTGGAGTTCAGACGTGTGCTCTTCCGATCT**CTAATTTTTTTTCTTTCTCCC** |  |  |
| 15 | ihhb-F | ACACTCTTTCCCTACACGACGCTCTTCCGATCT**GAGGGAAAATTAAAAATAAGGTG** | 376 | 52 |
|  | ihhb-R | GTGACTGGAGTTCAGACGTGTGCTCTTCCGATCT**CCATAAAACCATATAAAAACTC** |  |  |
| 16 | slco4a1-F | ACACTCTTTCCCTACACGACGCTCTTCCGATCT**TTTGTAATTAAAAGGAAATGG** | 436 | 54 |
|  | slco4a1-R | GTGACTGGAGTTCAGACGTGTGCTCTTCCGATCT**TAATTACACAACAAACACAACTC** |  |  |
| 17 | ephb2a-F | ACACTCTTTCCCTACACGACGCTCTTCCGATCT**TTTTTTGTATGGGGGTTTTA** | 362 | 52 |
|  | ephb2a-R | GTGACTGGAGTTCAGACGTGTGCTCTTCCGATCT**CTCTTTTATATCTAACCATTTTTC** |  |  |
| 18 | BX324216.3-F | ACACTCTTTCCCTACACGACGCTCTTCCGATCT**GGTTTAGTTGTTGTTTTAATTT** | 397 | 52 |
|  | BX324216.3-R | GTGACTGGAGTTCAGACGTGTGCTCTTCCGATCT**TTTCACATTTTCTCTCAATAACC** |  |  |
| 19 | rpa1-F | ACACTCTTTCCCTACACGACGCTCTTCCGATCT**AGTTAATAGTAAAGAGAATGAGG** | 479 | 52 |
|  | rpa1-R | GTGACTGGAGTTCAGACGTGTGCTCTTCCGATCT**ACAAAACAATACAACACAAAAA** |  |  |
| 20 | ostm1-F | ACACTCTTTCCCTACACGACGCTCTTCCGATCT**GTTGTAGGGTTTTTTAAGTT** | 335 | 52 |
|  | ostm1-R | GTGACTGGAGTTCAGACGTGTGCTCTTCCGATCT**CAAAAATTAAACCTAACTTCC** |  |  |

Table S2: General mapping statistics and bisulfite conversion efficiency. Presented are total number of sequences (150 base pairs, paired end), mapping efficiency (Map Eff), bisulfite conversion efficiency (Bis Eff), number of cytosines in CpG context covered (cov) with 1 read (1r), 5 reads (5r) and 10 reads (10r), coverage %CpG methylation based on cytosines with at least 5 reads (%mC in CpG)

|  | control 1 | control 2 | control 3 | exposed 1 | exposed 2 | exposed 3 | Average |
| --- | --- | --- | --- | --- | --- | --- | --- |
| Seq pairs (mln) | 122 | 125 | 124 | 127 | 126 | 138 | 127 |
| Map Eff (%) | 73.9 | 75.4 | 74.7 | 75.6 | 76.4 | 76.6 | 75 |
| Bis Eff (%) | 99.7 | 99.8 | 98.8 | 95.6 | 99.7 | 99.8 | 98 |
| Cov (1r) | 43009789 | 42772154 | 43209844 | 43549449 | 42887509 | 44183288 | 43268672 |
| Cov (5r) | 23678001 | 24024465 | 24472627 | 25883116 | 23399883 | 28766292 | 25037397 |
| Cov (10r) | 6382883 | 8054151 | 7117260 | 7725439 | 6760148 | 9465872 | 7584292 |
| %mC in CpG | 80.2 | 81.6 | 80.6 | 80.9 | 80.6 | 80.4 | 81 |

Table S3: IPA analysis of developmental DMRs and transcriptional start sites. Top 20 upstream regulators from ingenuity pathway analysis from differentially methylated regions (DMRs) located at developmental DMRs and around transcriptional start sites (+/- 2kb).

| developmental DMRs | | Transcriptional start sites | | |
| --- | --- | --- | --- | --- |
| Upstream Regulator | p-value of overlap | Upstream Regulator | p-value of overlap |  |
| FSH | 4,25E-05 | ST1926 | 3,08E-06 |  |
| TGFB1 | 4,31E-05 | TP53 | 2,08E-05 |  |
| PDGFC | 5,21E-05 | CD 437 | 6,72E-05 |  |
| DMP1 | 6,18E-05 | ESR1 | 6,76E-05 |  |
| AGT | 1,42E-04 | beta-estradiol | 1,71E-04 |  |
| tretinoin | 1,71E-04 | TGFB1 | 2,47E-04 |  |
| beta-estradiol | 1,82E-04 | DMD | 3,49E-04 |  |
| GLI1 | 2,00E-04 | SMAD4 | 3,57E-04 |  |
| FOXF2 | 2,07E-04 | arsenic trioxide | 4,12E-04 |  |
| ERBB3 | 2,48E-04 | ABL1 | 4,42E-04 |  |
| 1,2-dithiol-3-thione | 2,67E-04 | mir-27 | 4,51E-04 |  |
| cyclohexanecarboxylic acid | 3,00E-04 | HNF4A | 5,36E-04 |  |
| miR-21-5p (and other miRNAs w/seed AGCUUAU) | 3,17E-04 | androgen | 6,06E-04 |  |
| VEGFA | 3,58E-04 | MYCN | 6,53E-04 |  |
| GNAQ | 3,65E-04 | mono-(2-ethylhexyl)phthalate | 7,21E-04 |  |
| NREP | 4,18E-04 | HP1 | 7,79E-04 |  |
| miR-143-3p (and other miRNAs w/seed GAGAUGA) | 4,18E-04 | incyclinide | 7,79E-04 |  |
| SRF | 5,40E-04 | FOXO3 | 9,54E-04 |  |
| ESR1 | 5,47E-04 | SIAH2 | 9,64E-04 |  |
| PROM1 | 5,97E-04 | HIF1A | 1,03E-03 |  |

Table S4: Genomic locations of the predicted motifs by MEME. Shown are the location (chr:start-end) and similarity of the site with the respective motif (p-value).

| Motif 1 | | Motif 2 | | Motif 3 | |  |
| --- | --- | --- | --- | --- | --- | --- |
| Genomic location | p-value | Genomic location | p-value | Genomic location | p-value | |
| 1:9611126-9612375 | 7.40E-20 | 10:4924501-4925875 | 3.40E-17 | 4:30662001-30663500 | 5.25E-19 | |
| 16:30645376-30646750 | 7.40E-20 | 4:11065501-11066750 | 5.72E-16 | 4:60912626-60914000 | 5.25E-19 | |
| 23:38874751-38876125 | 7.40E-20 | 15:34209626-34210875 | 2.32E-14 | 4:49428876-49430125 | 4.21E-18 | |
| 6:39703126-39704500 | 2.07E-19 | 17:24817876-24819125 | 2.71E-14 | 4:53338126-53339500 | 8.35E-18 | |
| 5:50433501-50434875 | 8.35E-19 | 19:9231876-9233125 | 1.47E-13 | 4:62867751-62869125 | 1.17E-17 | |
| 23:33784251-33785500 | 1.38E-18 | 15:19862751-19864000 | 1.64E-13 | 4:33435626-33436875 | 1.17E-17 | |
| 15:34209626-34210875 | 1.51E-18 | 3:25887376-25888625 | 1.83E-13 | 4:50923126-50924500 | 1.45E-17 | |
| 17:24817876-24819125 | 2.58E-18 | 16:30645376-30646750 | 1.83E-13 | 4:32859251-32860625 | 1.62E-17 | |
| 2:8891376-8892625 | 1.59E-17 | 6:39703126-39704500 | 2.28E-13 | 4:32819501-32820875 | 6.56E-17 | |
| 2:30929876-30931250 | 2.53E-17 | 5:56769376-56770625 | 2.28E-13 | 15:902501-903875 | 2.09E-14 | |
| 11:28751-30000 | 3.52E-17 | 1:9611126-9612375 | 4.77E-13 | 4:63622876-63624250 | 2.91E-14 | |
| 24:24126626-24127875 | 4.76E-17 | 24:24126626-24127875 | 5.85E-13 | 25:35780126-35781500 | 2.81E-10 | |
| 15:19862751-19864000 | 7.34E-17 | 2:8891376-8892625 | 1.28E-12 |  |  | |
| 16:10372376-10373625 | 7.81E-17 | 23:36320501-36321750 | 2.39E-12 |  |  | |
| 16:42866126-42867500 | 1.88E-16 | 11:2787376-2788750 | 2.61E-12 |  |  | |
| 3:29557376-29558750 | 4.48E-16 | 23:33784251-33785500 | 2.84E-12 |  |  | |
| 21:22673501-22674750 | 8.27E-16 | 8:25586001-25587250 | 3.95E-12 |  |  | |
| 8:25586001-25587250 | 3.34E-15 | 16:10372376-10373625 | 4.64E-12 |  |  | |
|  |  | 16:42866126-42867500 | 7.98E-12 |  |  | |
|  |  | 23:38874751-38876125 | 2.04E-11 |  |  | |
|  |  | 13:11911251-11912625 | 2.18E-11 |  |  | |
|  |  | 11:28751-30000 | 2.49E-11 |  |  | |
|  |  | 2:30929876-30931250 | 4.19E-11 |  |  | |
|  |  | 22:19381001-19382250 | 9.17E-11 |  |  | |

Figure S1: Differentially methylated regions for BisPCR2 analysis. Shown is data derived from Seqmonk with the gene track (mRNA, upper part), and in each of the treatment tracks, the read depth (blue are unmethylated Cs and red are methylated Cs) and the methylation levels represented as bar graphs. Highlighted is the differentially methylated region as assessed by methylKit. Error bars represent SEM.

| *fbxo46*  ** |
| --- |
| *myo1c*  ** |
| *sacs* |
| **  *ccdc25* |
| **  *atm*  ** |
| *epx* |
| **  *marcksa*   |
| *tpd52* |
| **  *pak2b* |
| **  *crk* |
| **  *sox7* |
| **  *sod3a* |
| **  *fgf2* |
| **  *kcnd3* |
| **  *ihhb* |
| **  *slco4a1* |
| **  *ephb2a* |
| **  *BX324216.3* |
| **  *rpa1* |
| **  *ostm1*  ** |

Figure S2: Standard curve analysis of all targets by BisPCR2. Calibration curves show the theoretical % methylation from the standard curve versus the observed methylation as assessed by BisPCR.

Figure S3: Density plots of different histone marks and accessible chromatin at sphere stage around differentially methylated regions (DMRs), spanning a region of 10 kb.


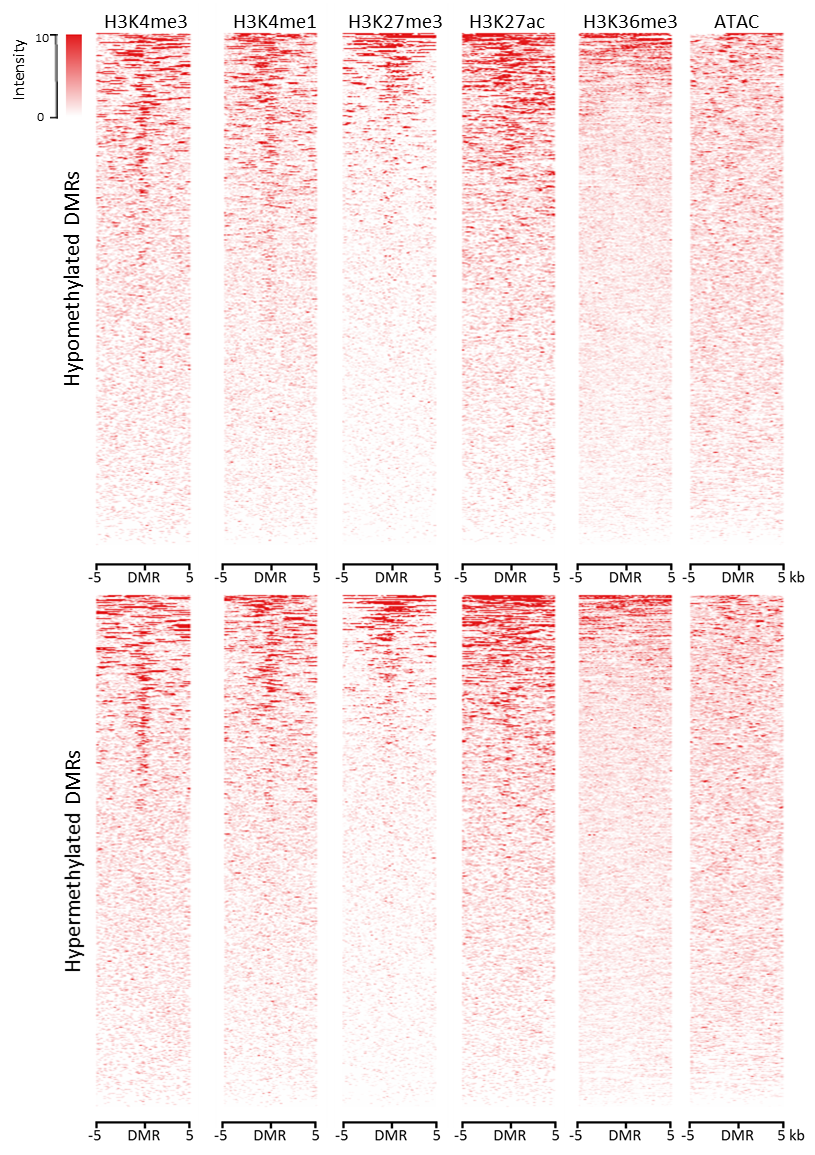


Figure S4: Relationship between gene expression and high content GC promoter DNA methylation. Correlation plot between log2 gene expression versus % DNA methylation located at high content GC promoters.

Figure S5: Background genomic clustering analysis of all measured methylation tiles. Circular plots showing overlap in overrepresented clusters of differentially methylated regions in blue and background methylation clusters in black.


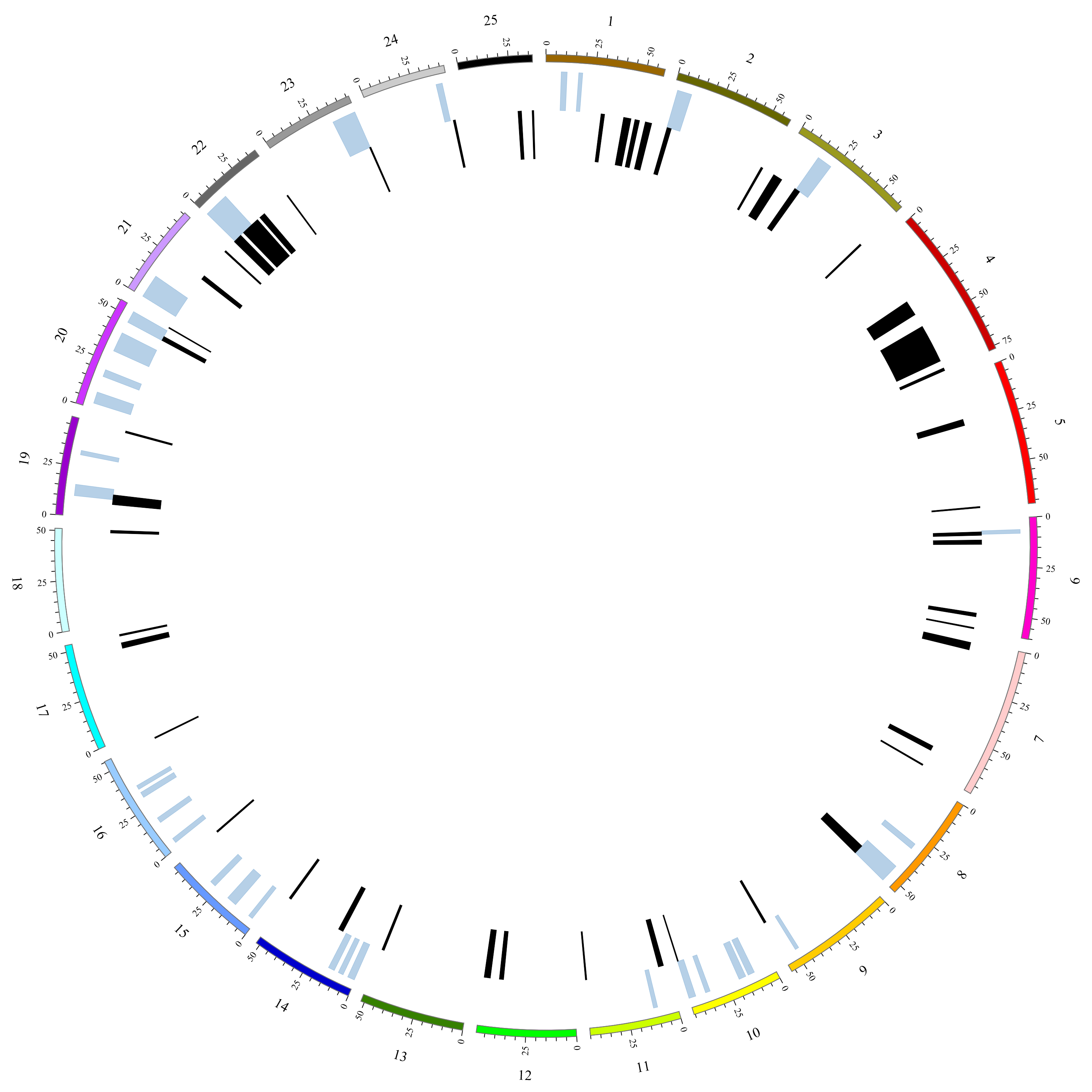


Figure S6: Background genomic clustering analysis of annotated genes. Circular plots showing overlap in overrepresented clusters of differentially expressed genes in red and background gene clusters in black.


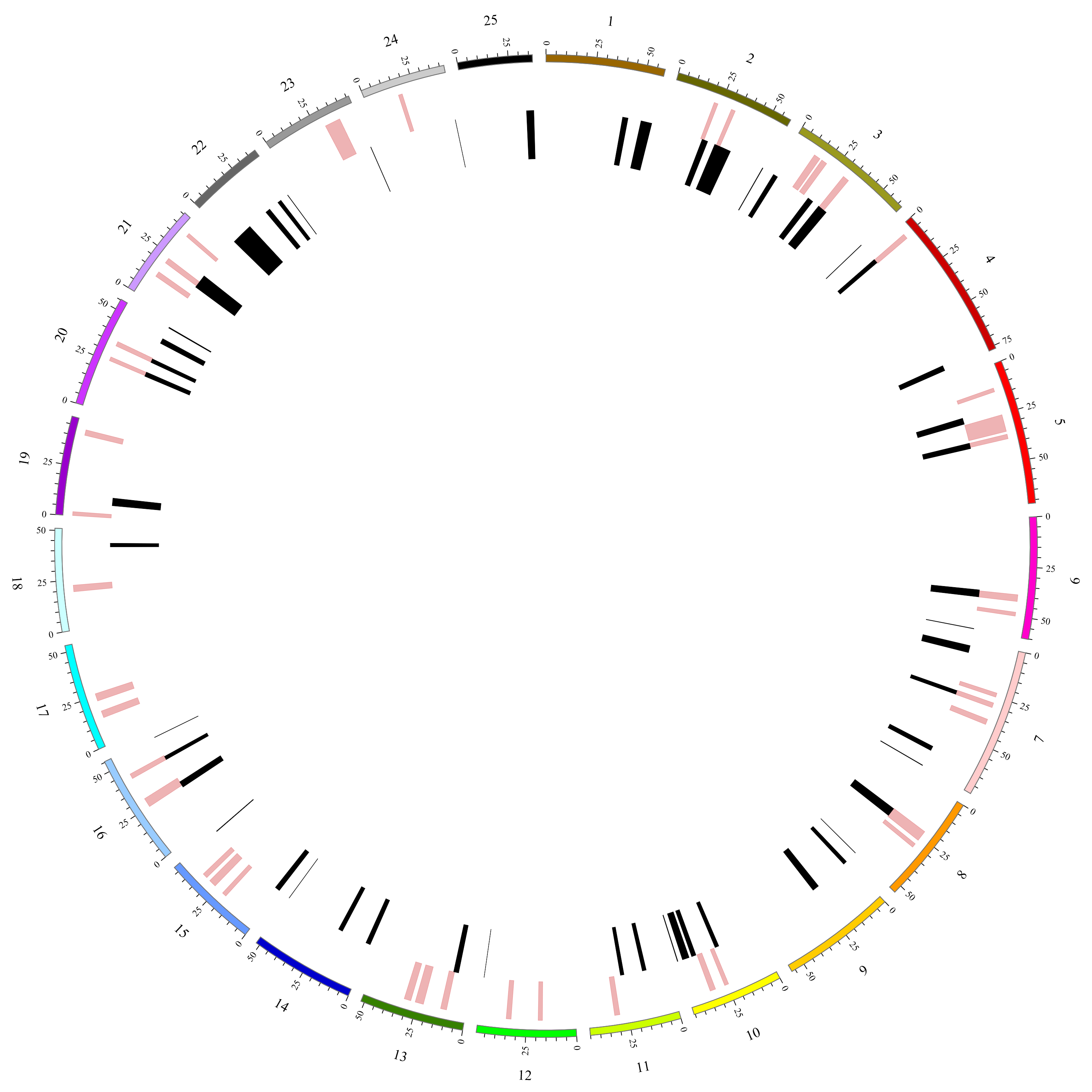

Figure S7: Correlation between WGBS and BisPCR2. XY-scatter plot of the methylation difference between control and exposed samples following BisPCR2 and WGBS analysis. In colours are loci that showed no difference with BisPCR2 compared to WGBS.

1. Bernstein, D. L., Kameswaran, V., Le Lay, J. E., Sheaffer, K. L. & Kaestner, K. H. The BisPCR2 method for targeted bisulfite sequencing. *Epigenetics Chromatin* **8,** 27 (2015).

2. Kamstra, J. H., Sales, L. B., Aleström, P. & Legler, J. Differential DNA methylation at conserved non-genic elements and evidence for transgenerational inheritance following developmental exposure to mono(2-ethylhexyl) phthalate and 5-azacytidine in zebrafish. *Epigenetics Chromatin* **10,** 20 (2017).
